# Tri‐Functional Electrocatalysis for Zinc–Air Battery Coupled Water Splitting: A Catalyst‐Electrode‐Device‐System Perspective

**DOI:** 10.1002/advs.77619

**Published:** 2026-09-10

**Authors:** Xixi Di, Chao Duan, Shuzhen Li, Yiyang Che, Xiaoshuang Liu, Chuanyin Xiong, Mengxia Shen, Yichao Wang, Derek Hao, Yonghao Ni

**Affiliations:** ^1^ College of Bioresources Chemical and Materials Engineering Shaanxi University of Science and Technology Xi'an China; ^2^ School of Science RMIT University Melbourne Australia; ^3^ Limerick Pulp and Paper Centre and Department of Chemical Engineering University of New Brunswick Fredericton New Brunswick Canada

**Keywords:** self‐powered systems, tri‐functional electrocatalysts, water splitting, zinc–air batteries

## Abstract

Tri‑functional electrocatalysis is crucial for coupling rechargeable zinc‑air batteries with water splitting (WS), enabling self‑powered hydrogen production and versatile energy conversion. Although numerous catalysts demonstrate excellent half‐cell activities toward the hydrogen evolution reaction, oxygen evolution reaction, and oxygen reduction reaction, their practical application in battery‐driven water electrolysis remains challenging. The major bottleneck is multiscale mismatch, which involves conflicting adsorption energetics at atomic sites, unstable gas‑liquid‑solid interfaces at electrodes, device degradation under alternating charge‑discharge and electrolysis modes, and voltage‑power imbalances at the system level. This review goes beyond conventional material summaries by constructing a catalyst–electrode–device–system framework to systematically examine how electronic modulation, defect engineering, heterostructure design, single‐atom site tuning, and self‐supported electrode construction influence intrinsic activity, mass transport, interfacial durability, and overall cell performance. Importantly, the actual operating point of the coupled system is determined by the intersection of the battery discharge curve and the electrolyzer polarization curve, rather than by isolated half‑cell metrics alone. Finally, we highlight future opportunities including operando characterization techniques, AI‑driven catalyst screening, standardized device evaluation protocols, and system‐level co‑design for practical renewable energy devices.

## Graphical abstract

This review establishes a catalyst–electrode–device–system framework for tri‐functional HER/OER/ORR electrocatalysis that couples water splitting with Zn–air batteries. Five catalyst families are covered: noble‐metal, metal‐based, single‐atom, heterostructure, and metal‐free systems. It analyzes how electronic modulation, defect engineering, and interface design influence catalytic activity, mass transport, and durability, thereby offering guidance for the rational design of practical renewable energy devices.

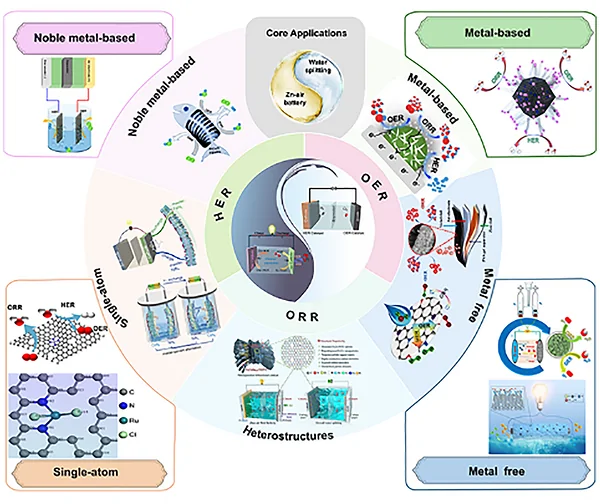

## 1 Introduction

The global transition toward carbon neutrality is accelerating the development of efficient and sustainable technologies for energy conversion, storage, and clean fuel production [1, 2, 3]. Among the various electrochemical energy systems, water electrolysis for hydrogen production and rechargeable zinc–air batteries (ZAB) represent key directions for clean fuel production and high‐energy‐density energy storage, respectively [4, 5, 6, 7, 8]. Water electrolysis can convert intermittent renewable electricity into high‐energy‐density hydrogen, and it is considered one of the key technologies for building a future hydrogen economy [2, 9, 10, 11]. ZAB uses zinc metal as the anode and oxygen from the air as the cathode reactant, offering advantages such as high theoretical energy density, good safety, abundant raw materials, and environmental friendliness [12, 13, 14], and thus holds broad application prospects in large‐scale energy storage, portable electronic devices, and flexible wearable systems.

However, large‐scale application of these two types of systems remains limited by slow reaction kinetics and insufficient catalyst stability. In the water splitting (WS) process, the cathodic hydrogen evolution reaction (HER) and the anodic oxygen evolution reaction (OER) involve proton/water molecule activation, hydrogen intermediate adsorption, and multistep oxygen intermediate transformation, respectively [15, 16, 17]. In ZAB, the air electrode undergoes oxygen reduction reaction (ORR) and OER during discharge and charge, respectively [13, 14, 18]. HER, OER, and ORR are all typical multi‐electron transfer reactions with complex kinetic processes, placing high demands on the electronic structure of the catalyst, reactant adsorption strength, interfacial hydrophilicity or hydrophobicity, and its capabilities for charge and mass transport [19, 20, 21]. More importantly, the optimal active sites and reaction environments for these three reactions are not entirely consistent. For example, HER typically requires a moderate H* (* is the active site) adsorption free energy; OER necessitates effective regulation of oxygen‐containing intermediates such as OH*, O*, and OOH*; while ORR requires simultaneous promotion of O_2_ activation and enhancement of the selectivity of the four‐electron reduction pathway [22, 23]. Therefore, how to balance HER, OER, and ORR activities within the same catalytic system is a core scientific challenge in the design of tri‐functional electrocatalysts.

Traditionally, highly efficient HER catalysts have primarily relied on Pt‐based materials, while OER catalysts have mostly utilized precious metal oxides such as IrO_2_ or RuO_2_, and ORR catalysts have been represented by Pt/C [24]. However, the high cost, resource scarcity, and long‐term operational stability issues associated with precious metal catalysts have limited their large‐scale application. To reduce reliance on precious metals, researchers have developed a variety of non‐precious metal‐based catalysts, including transition metal oxides, hydroxides, sulfides, phosphides, nitrides, carbides, metal–organic framework (MOF) derivatives, heteroatom‐doped carbon materials, and single‐atom catalysts [25, 26, 27]. By modulating electronic structures, increasing active site density, constructing heterojunctions, and optimizing pore structures, these materials demonstrate excellent performance in HER, OER, or ORR [28]. These catalysts enable rechargeable ZABs and water‐splitting devices to operate on a shared material platform, providing a basis for the development of ZAB‐driven self‐powered water‐splitting systems [29].

Over the past decade, the appeal and booming trend of tri‐functional electrocatalysts in the fields of ZAB and WS have been fully reflected in the dense co‑occurrence network diagram of related keywords and the rapid growth in the number of publications (Figure 1a,b). Up to now, a large number of studies have reported optimizing material composition, controlling morphology, designing heterostructures, defect engineering, and constructing single‐atom sites, thereby substantially expanding the range of material systems and design strategies for tri‐functional catalysts (Figure 1c) [19, 20, 30, 31, 32, 33]. For example, metal/nitrogen co‐doped carbon materials can enhance ORR activity by adjusting the charge distribution of the carbon framework and the metal coordination environment [34, 35]. Transition metal phosphides, sulfides, and nitrides demonstrate excellent catalytic potential in HER and OER [19]. Heterostructures can optimize the adsorption of key intermediates through interfacial charge redistribution, and single‐atom catalysts can maximize metal utilization and regulate coordination structures at the atomic scale [36, 37, 38]. These advancements have driven the evolution of tri‐functional electrocatalysts from the optimization of individual properties toward synergistic catalysis across multiple reactions.

**FIGURE 1:**
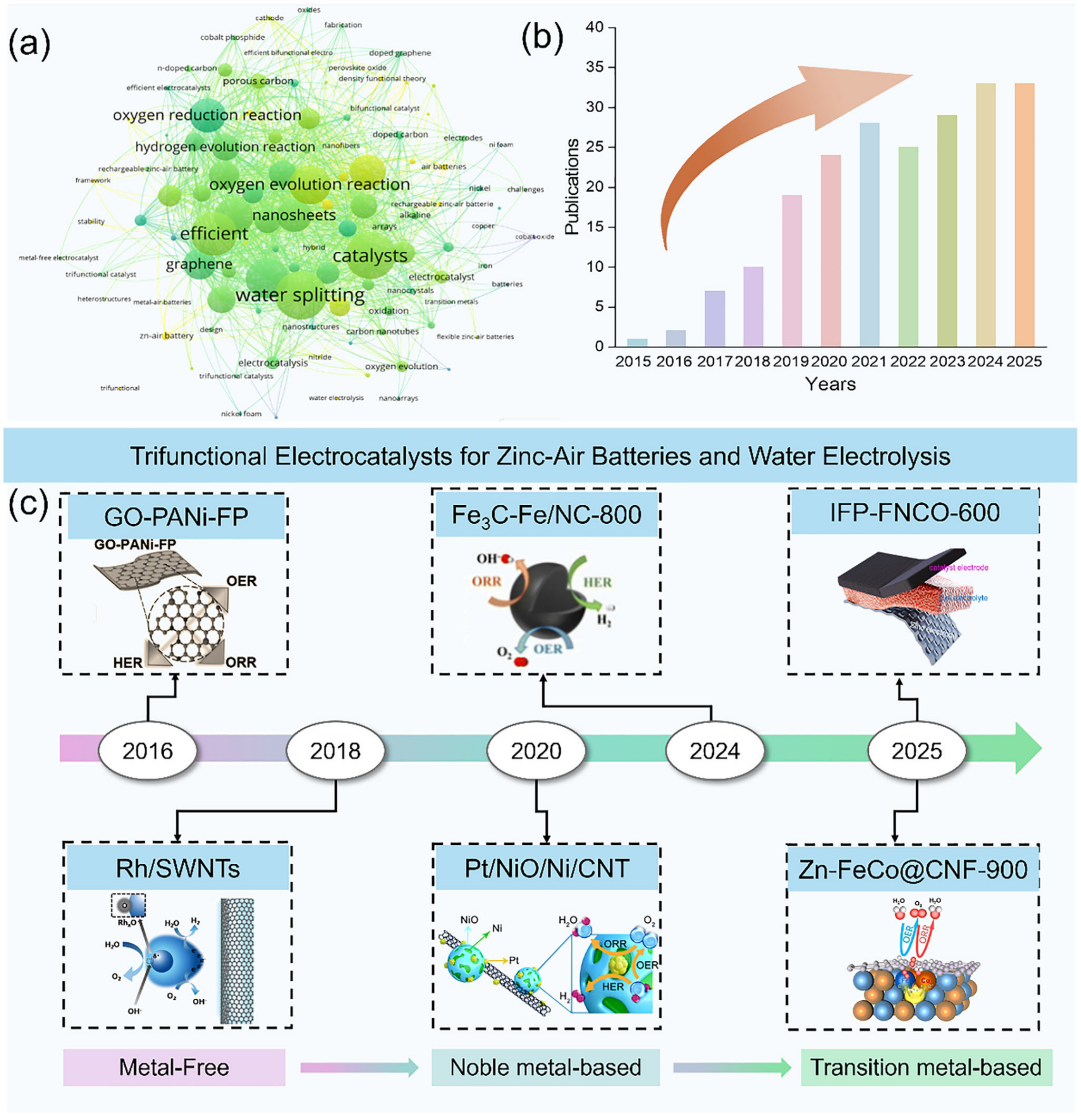
(a) Network diagram of keywords related to tri‐functional electrocatalysts for ZAB and water electrolysis. (b) Number of publications based on synergistic composite catalysts from 2015 to 2025. (c) Research and development of tri‐functional electrocatalysts: N, P, F‐tridoped graphene (Reproduced with permission [30]. Copyright 2016, Wiley‐VCH); Rh/SWNTs (Reproduced with permission [31]. Copyright 2018, ACS); Pt/NiO/Ni/ CNT (Reproduced with permission [32]. Copyright 2020, RSC); Fe‐based 3D framework (Reproduced with permission [20]. Copyright 2024, RSC); IFP‐FNCO‐600 (Reproduced with permission [33]. Copyright 2025, ACS) and Zn‐doped FeCo alloy (Reproduced with permission [19]. Copyright 2025, Elsevier).

Many studies primarily employ three‐electrode systems or rotating disk electrodes (RDEs) to assess the intrinsic activity of catalysts, investigating metrics such as HER/OER overpotentials, ORR half‐wave potential, Tafel slope, and stability test duration [9]. While these half‐cell‐derived parameters are essential for understanding the intrinsic activity of materials, they are inadequate for predicting catalyst performance in actual devices [39, 40]. Therefore, excellent half‐cell performance does not necessarily imply excellent device performance.

This challenge becomes even more pronounced when ZABs are coupled with water electrolysis systems. In rechargeable ZAB, the air electrode must remain stable under alternating conditions of discharge ORR and charge OER, during which the catalyst may undergo cyclic redox reactions, carbon support corrosion, active phase restructuring, and three‐phase interface degradation [39, 41, 42, 43]. For water electrolysis systems, the cathode HER and anode OER must operate stably over extended periods in the same electrolyte while withstanding effects such as bubble scouring, electrolyte corrosion, and local pH fluctuations [26, 44, 45, 46]. When ZABs are further utilized to drive water electrolysis for hydrogen production, system performance no longer depends solely on the overpotential of a single catalyst but on the dynamic matching between the discharge curve of the ZAB and the polarization curve of the electrolyzer [47, 48]. Voltage plateaus, output power, capacity decay, load variations, system energy efficiency, and long‐term operational stability all become key factors determining the performance of self‐powered hydrogen production systems.

In other words, a multiscale cognitive framework should be established, spanning from active sites at the atomic scale, structural regulation at the nanoscale, and electrode construction at the mesoscale, to device performance and system energy flow management at the macroscale. Within this framework, catalyst design must not only optimize the energy barriers of adsorption intermediates for HER, OER, and ORR, but also consider electrode conductivity, pore connectivity, stability at gas/liquid/solid triple interfaces, dynamic restructuring behavior, and matching of system operating points. While these advances are impressive, most existing reviews focus on material synthesis and half‑cell performance, with limited attention to the gap between laboratory‑scale activity and device‑level practicality. Moreover, the integration of ZABs with water splitting introduces system‑level challenges that are rarely discussed in a unified framework.

In this context, this review systematically reviews tri‐functional electrocatalysts, including their material design, preparation methods, catalytic mechanisms, device applications, and system integration. First, this review outlines the reaction mechanisms, key intermediates, and performance evaluation metrics for HER, OER, and ORR, and analyzes the common requirements and inherent contradictions in the design of tri‐functional catalysts. Subsequently, it focuses on the optimization strategies for tri‐functional catalysts, encompassing the synthesis methods and corresponding tuning strategies associated with two pathways: electronic regulation and structural engineering. Next, the review discusses the structural features, structure–property relationships, and reaction mechanisms of tri‐functional catalysts categorized by type, including non‐metal‐based, noble metal‐based, transition metal‐based, and single‐atom catalysts.

## 2 Design Strategies and Synthesis Methods for Tri‐Functional Catalysts

### 2.1 Reaction Mechanisms of HER, OER and ORR

HER involves two‐electron transfer (Figure 2a). The metal‐H bond strength critically affects activity: it must be strong enough to promote H* adsorption (Volmer reaction) as Equations (1) and (2), but not too strong to hinder H* desorption and cause active site poisoning.

**FIGURE 2:**
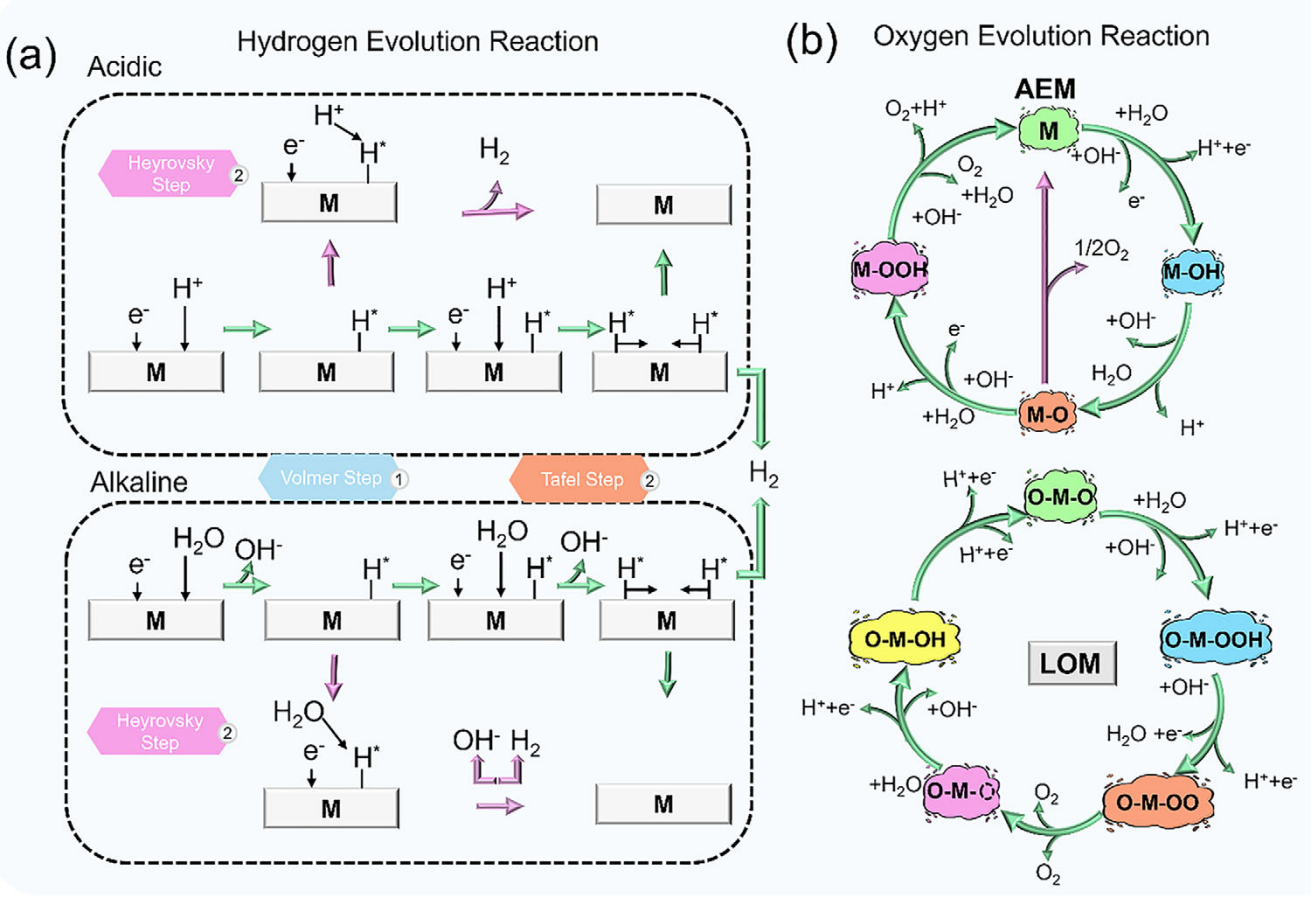
(a) Schematic mechanism of hydrogen evolution on the surface of an electrode under acidic (top) and alkaline (bottom) conditions. (b) Schematic illustration of the AEM (top) and LOM (bottom) for the OER, where “M” represents the active site.

According to the Sabatier principle [49, 50], ideal catalysts achieve balanced hydrogen adsorption/desorption. Too strong or too weak binding hinders intermediates and H_2_ formation. In acidic media, protons come from hydrated hydrogen ions; in basic media, from H_2_O. HER proceeds via two pathways: low H* coverage leads to the Heyrovsky reaction as Equations (3) and (4); high coverage leads to the Tafel reaction as Equation (5). The hydrogen adsorption free energy (Δ*G*_H*_) is a key descriptor; values near zero indicate superior performance.

Volmer reaction:

(1)

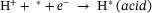



(2)

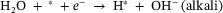




Heyrovsky reaction:

(3) $$H^{*}+H^{+}+e^{-}\to H_{2}\left(\mathrm{acid}\right)$$

(4) $$H^{*}+H_{2}O+e^{-}\to^{*}+OH^{-}+H_{2}\left(\mathrm{alkali}\right)$$

Tafel reaction:

(5) $$2H^{*}\to^{*}+H_{2}$$

Unlike HER (two‐electron transfer), OER at the anode is a complex four‐electron‐proton cooperative transfer process [51]. Its high overpotential and kinetic barrier make it the bottleneck limiting overall efficiency of water electrolysis for hydrogen production [52]. The reaction mechanism in electrolytes with different pH are shown in Equations (6)–(13):

OER in acidic electrolyte:

(6) $$H_{2}O{+}^{*}\to HO^{*}+H^{+}+e^{-}$$

(7) $$HO^{*}\to O^{*}+H^{+}+e^{-}$$

(8) $$O^{*}+H_{2}O\to \mathrm{HO}O^{*}+H^{+}+e^{-}$$

(9) $$\mathrm{HO}O^{*}\to^{*}+O_{2}+H^{+}+e^{-}$$

OER in alkaline electrolyte:

(10) $$OH^{-}{+}^{*}\to HO^{*}+e^{-}$$

(11) $$HO^{*}+OH^{-}\to O^{*}+H_{2}O+e^{-}$$

(12) $$O^{*}+OH^{-}\to \mathrm{HO}O^{*}+e^{-}$$

(13)

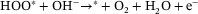




The OER mechanisms currently accepted: adsorbate evolution mechanism (AEM) and the lattice oxygen mechanism (LOM). According to the AEM (Figure 2b), the evolved oxygen molecules originate from water molecules adsorbed on the catalyst surface [49, 53]. In the initial stage, water adsorption produces hydroxyl and oxygen intermediates; O─O bond formation is rate‐limiting; tuning hydroxyl free energy enhances stability. Identifying the dominant OER mechanism matters because AEM and LOM impose distinct trade‑offs between activity and stability, directly influencing the design strategy for balanced tri‐functional catalysis. According to the LOM, lattice oxygen participates; oxygen originates from lattice oxygen and water; breaks the scaling relationship; is pH‐dependent; but lattice oxygen evolution causes structural collapse; protective layers or doping are used to maintain stability [54, 55, 56].

ORR is the reverse process of OER with multi‐electron transfer [57, 58]. It follows a direct 4e^−^ pathway (H_2_O or OH^−^) or an indirect 2e^−^ pathway (H_2_O_2_ or HO_2_^−^) as in Equations (14)–(19). The 4e^−^ selectivity is key. Kinetics are slow; the first electron transfer is rate‐determining, strongly affected by adsorption of oxygen intermediates. Catalysts with moderate binding energy for oxygen species can accelerate ORR and reduce overpotential.

In alkaline media:

(14) $$O_{2}+2H_{2}O+4e^{-}\to 4OH^{-}$$

(15) $$O_{2}+2H_{2}O+2e^{-}\to H{O_{2}}^{-}+OH^{-}$$

(16) $$H{O_{2}}^{-}+2H_{2}O+2e^{-}\to 3OH^{-}$$

In acidic media:

(17) $$O_{2}+4H^{+}+4e^{-}\to 2H_{2}O$$

(18) $$O_{2}+2H^{+}+2e^{-}\to H_{2}O_{2}$$

(19) $$H_{2}O_{2}+2H^{+}+2e^{-}\to 2H_{2}O$$

These three equations are the core electrochemical processes in ZAB (ORR/OER) and WS (HER/OER); therefore, the design of a tri‐functional catalyst must address the requirements of all three simultaneously.

### 2.2 Preparation Method and Modification Strategy for a Tri‐Functional Electrocatalyst

The performance optimization of tri‐functional electrocatalysts essentially relies on the synergistic enhancement of intrinsic activity and structural characteristics. As shown in Figure 3, rational design follows two pathways: first, tuning the electronic structure to boost the intrinsic catalytic capability of active sites; second, creating an ideal reaction microenvironment through structural engineering to maximize active site utilization, facilitate mass transfer, and enhance stability [59, 60]. These two approaches complement each other.

**FIGURE 3:**
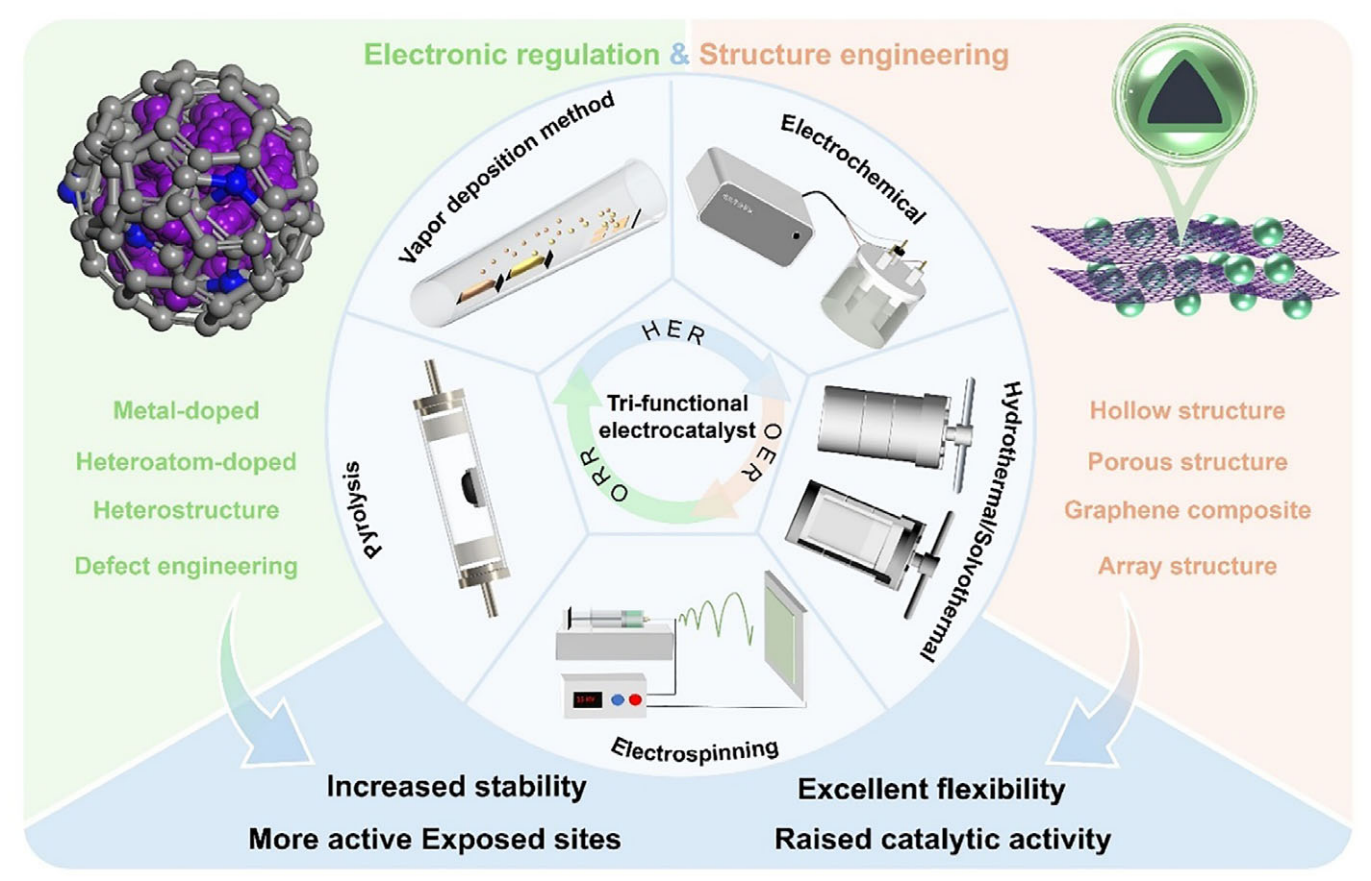
Preparation and applications of tri‐functional electrocatalysts.

Electronic regulation focuses on optimizing the adsorption strength of reaction intermediates on active sites. The key strategies include metal doping (e.g., introducing Fe, Co, Ni, Mn [20, 26, 61, 62, 63]) to adjust the d‑band center and overcome scaling relationships; such materials are often prepared by pyrolysis, for example, high‑temperature pyrolysis of metal‑organic precursors under an inert atmosphere to efficiently obtain metal/nitrogen/carbon composites. Heteroatom doping (N, P, S, B [28, 64]) alters charge distribution on the carbon framework via electronegativity differences; pyrolysis is also the preferred method for preparing doped carbon materials. Heterostructures (Schottky junctions or p‑n junctions [65]) use built‑in electric fields at interfaces to optimize adsorption energies; they are typically fabricated by vapor deposition (precise atomic‑scale interface construction) or hydrothermal methods (growing heterointerfaces in sealed high‑pressure environments). Defect engineering (anion/cation vacancies, grain boundaries [29]) modifies local coordination environments; hydrothermal methods and electrochemical methods (e.g., anodization) are commonly used for controlled defect introduction.

An optimized electronic structure requires suitable macroscopic/microscopic morphology to amplify its performance. Structural engineering aims to construct an efficient physical framework, primarily involving multi‑scale pore structures (micro‑, meso‑, macropores), often realized via pyrolysis of precursors with sacrificial templates or phase separation. Hollow/core‑shell structures (nanospheres, nanotubes, core‐shell) provide internal cavities for additional reaction interfaces and volume buffering, while the shell prevents agglomeration or dissolution of active components; these are frequently synthesized by hydrothermal or template‑assisted pyrolysis methods. Carbon‑based composites combine with highly conductive carbon materials, where the carbon framework serves as a conductive matrix and provides abundant defects/doping sites; pyrolysis of carbon‑rich precursors (e.g., MOFs, polymers) is the most direct route [66, 67]. Array structures (vertically aligned nanowires/nanosheets on conductive substrates, or 3D interconnected porous networks) form binder‑free integrated electrodes, ensuring conductivity, mechanical robustness, and rapid transport pathways for reactants and bubbles. These are typically prepared by hydrothermal growth directly on substrates, or by electrochemical deposition to achieve strong substrate bonding and uniform loading. For flexible energy devices, electrospinning followed by heat treatment continuously produces one‑dimensional nanofiber mats [68] with high porosity and self‐supporting flexibility, representing a specialized structural engineering route rather than a standalone method category.

In summary, designing tri‐functional catalysts requires deep integration of electronic modulation (e.g., d‑band regulation, defect engineering) and structural engineering (e.g., porous architectures, self‑supporting networks). The appropriate synthesis method must be matched to the target design: pyrolysis for carbon‑based and doped materials, vapor deposition or hydrothermal methods for heterostructures and arrays, electrochemical methods for self‑supported electrodes, and electrospinning for flexible fiber membranes. Such strategic matching bridges the gap between intrinsic activity and device‑level performance. By doing so, we can accelerate the transition of high‑performance, highly stable, and low‑cost tri‐functional electrocatalysts from laboratory half‑cell studies to large‑scale applications, providing robust solutions for clean energy storage and conversion.

## 3 Electrocatalyst Types and Electrocatalytic Properties

The composition and structure of active sites both determine the intrinsic performance of catalysts. Hence, the representative systems covered in this chapter are divided into five types: metal‐free, noble‐metal‐based, transition‐metal‐based, heterojunction, and single‐atom catalysts. The subsequent sections systematically review their electrocatalytic properties, emphasizing the establishment of structure–activity relationships and the elucidation of catalytic mechanisms.

### 3.1 Metal‐Free Tri‐Functional Electrocatalyst

Research on metal‐free catalysts has expanded from single‑reaction to tri‐functional integration, and Table 1 summarizes representative metal‐free tri‐functional electrocatalysts reported in the literature in recent years. A landmark work by Zhang et al. [30] (Figure 4a) demonstrated that N, P, F‑tridoped graphene can simultaneously catalyze ORR, OER, and HER, and further assembled a Zn‑air‑driven WS system. This study established the paradigm of multi‑element doping to activate inert carbon frameworks. Its limitation, however, is that the roles of individual dopants remained speculative due to a lack of direct spectroscopic evidence, a gap that persisted for nearly a decade. Xu et al. [69] addressed this by using operando Raman to capture S─H bond vibrations, providing the first direct evidence that S atoms act as intrinsic active sites for HER/OER. DFT further revealed the N‑S co‑doping mechanism for Δ*G*_H*_ regulation. This work shifted the field from performance‑driven to mechanism‑driven research (Figure 4b). Yet two questions remain: (1) the study focused exclusively on S sites, leaving the role of N unclear; (2) the proposed site‑engineering guidelines have not been validated on other carbon matrices or doping configurations. Guided by such mechanistic insights, Muthurasu et al. [70] synthesized N, B, F‑doped porous carbon nanofibers via electrospinning, achieving an OER overpotential of 280 mV, comparable to metal oxides. DFT attributed this to electronic synergy: high‑electronegativity F induces charge redistribution, while B's vacant orbitals act as electron acceptors (Figure 4c). The porous structure (723 m^2^/g) boosted active site density and mass transport, raising hydrogen yield to 0.504 µL/s in a self‑powered system. The critical unresolved issue is the trade‑off: does optimizing OER compromise ORR or HER activity? The study did not systematically vary doping ratios to probe this. Pradhan et al. [71] extended non‐metallic catalysts to flexible solid‐state devices. Using a stepwise self‐assembly strategy, they constructed a g‐C_3_N_4_‐PPy/MOF‐derived carbon heterostructure (Figure 4d), achieving an ORR half‑wave potential of 0.92 V. The assembled solid‑state ZAB retained >79% capacity after bending from 0° to 180° and was integrated into a ring‑shaped wearable battery powering an LED. However, three caveats are noteworthy: (1) the 0.92 V was measured in liquid electrolyte, not in the solid‑state device; (2) long‑term cycling stability under mechanical deformation was only briefly reported; (3) the translation from a laboratory‑scale ring battery to practical wearables still faces hurdles in energy density, safety, and manufacturability. Beyond these individual studies, three systemic challenges persist: (1) The synergy of multi‑element doping remains empirically guided; no predictive theory exists for which heteroatom combinations work for which carbon host. (2) A unified model for tri‐functional active sites is absent; the structural requirements for ORR, OER, and HER are inherently conflicting on a pristine carbon framework. (3) Solid‑state catalytic mechanisms are unclear due to liquid‑cell studies, while flexible devices introduce rarely characterized triple‑phase interfaces. Future work must combine operando characterization with AI‑assisted screening to enable rational design and large‑scale deployment.

**TABLE 1:** Summary of some metal‐free tri‐functional electrocatalysts.

Material class	Catalyst	Doping elements	*E*_1/2_ORR (V)	*η*_10_OER (mV)	|Δ*E*| (V)^a^	*η*_10_HER (mV)	Power density (mW/cm^2^)	Specific capacity (mAh/g)	H_2_ evolution rate (µL/s)	Device validation	Refs.
Commercial benchmarks	Pt/C (20 wt.%)	/	0.88^b^	/	/	25^b^	/	/	/	Commercial	[72]
	IrO_2_	/	/	360^c^	/	/	/	/	/	Commercial	[73]
Metal‐free catalysts	GO‐PANi‐FP	N, P, F	/^b^	/^b^	/	520^b^	38^d^	/	0.496	Full device	[30]
	CNT‐550‐NS	N, S	0.81^b^	410^c^	0.40	95^c^	120^e^	781	/	Standalone device	[69]
	PCNFs	N, B, F	0.90^b^	280^c^	0.62	/	151.9^f^	729	0.504	Full device	[70]
	g‐C_3_N_4_‐PPy@CNR	N, B, F	0.92^b^	320^c^	0.60	/	125.23^f^	541.2	/	Full device	[71]

Measurement conditions:

^a^ Δ*E* = *E* **_{OER, j = 10}_ −** *E* **_{ORR, 1/2}_** [74].

^b^ 0.1 m KOH.

^c^ 1 m KOH.

^d^ 6 m KOH.

^e^ 6 m KOH + 0.2 m Zn(Ac)_2_.

^f^ 6 m KOH + 0.2 m Zn(CH_3_COO)_2_.

Full device: The catalyst has been tested in an integrated ZAB‐driven water splitting system. Standalone device: The catalyst has been tested in a ZAB or electrolyzer separately, but the coupled system data are missing.

**FIGURE 4:**
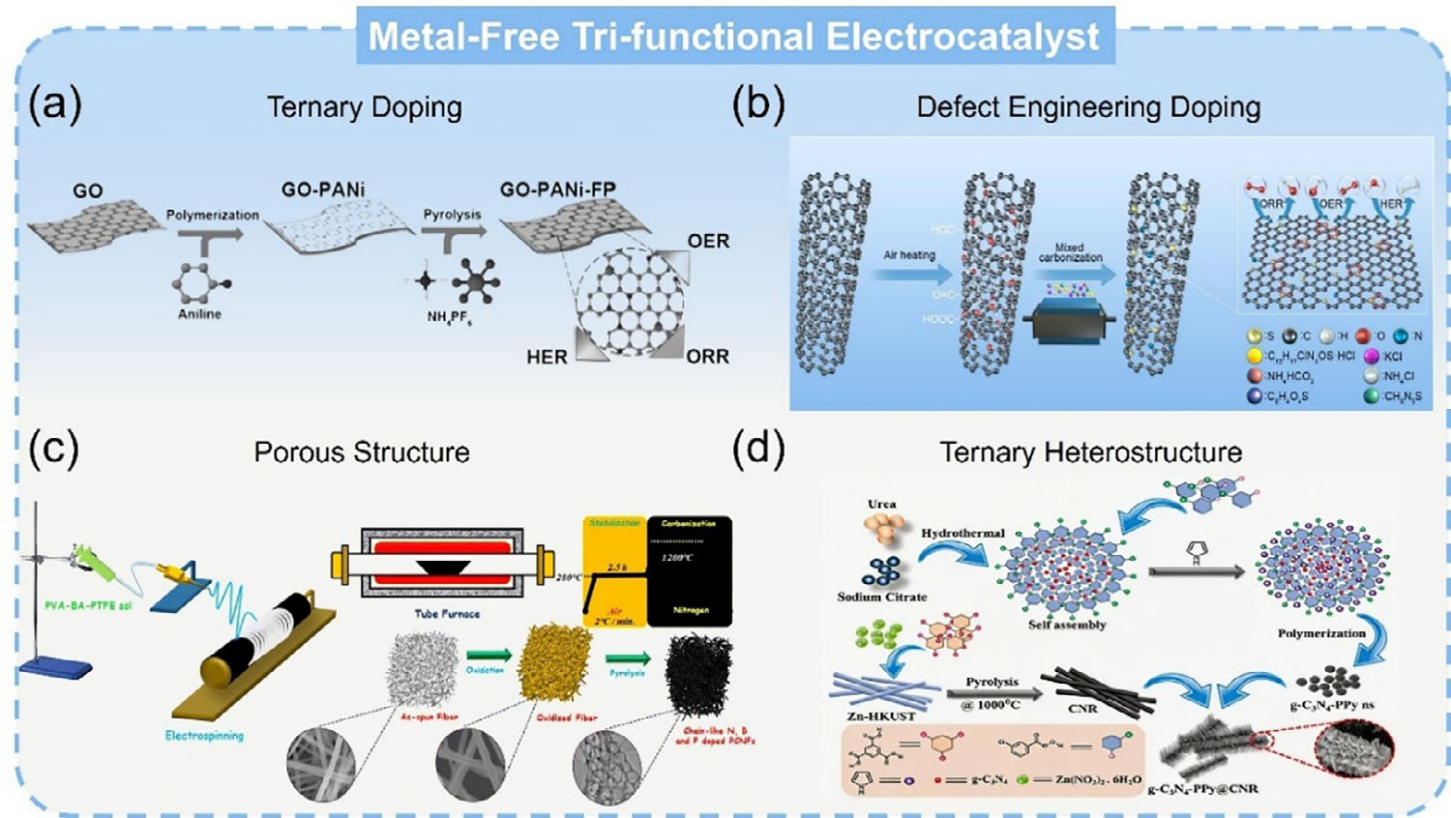
Metal‐free tri‐functional electrocatalyst: (a) Ternary doping. Reproduced with permission [30]. Copyright 2016, Wiley. (b) Defect engineering doping. Reproduced with permission [69]. Copyright 2023, Elsevier. (c) Porous structure. Reproduced with permission [70]. Copyright 2023, RSC. (d) Ternary heterostructure. Reproduced with permission [71]. Copyright 2025, Elsevier.

### 3.2 Noble Metal‐Based Tri‐Functional Electrocatalyst

Precious metal‐based materials (Pt, Ir, Ru, etc.) excel in single reactions but struggle to achieve tri‐functionality in one system. Their high cost and scarcity further limit large‐scale use. To address this, multi‐component strategies have been developed to regulate electronic structures and construct synergistic active sites. Based on design principles, current precious metal tri‐functional electrocatalysts fall into four categories: alloys/heterostructures, noble metal phosphides, oxide/chalcogenide heterojunctions, and nanoparticle/single‐atom hybrid systems, and Table 2 summarizes representative noble metal‐based tri‐functional electrocatalysts reported in the literature in recent years.

**TABLE 2:** Summary of some noble metal‐based tri‐functional electrocatalysts.

Classification	Catalyst	Noble metal loading	*E*_1/2_ORR (V)	*η*_10_OER (mV)	Δ*E* (V)^a^	*η*_10_HER (mV)	Power density (mW/cm^2^)	Specific capacity (mAh/g)	Device validation	Refs.
Commercial benchmarks	Pt/C (20 wt.%)	/	0.88^b^	/	/	25^c^	/	/	Commercial	[72]
	IrO_2_	/	/	360^c^	/	/	/	/	Commercial	[73]
Alloys and heterostructures	PtCo/Ir FBNWs	Pt = 8.4 µg/cm^2^ Ir = 1.98 µg/cm^2^	/^b^	426^c^	/	36^c^	/^d^	/	Full device	[75]
	Rh_6_Cu_1_ NCs	Pt = 85.8% Cu = 85.8%	0.85^b^	300^c^	0.68	36^c^	142.6^d^	804.3	Standalone device	[76]
	NF@Pt‐1	Pt = 0.7%	/	/^c^	/	32.8^c^	110^e^	665	Standalone device	[77]
Noble metal phosphides	Rh*_x_* P/NPC RuP/NPC	Rh = 0.4% Rh = 0.5%	0.89^b^ 0.89^b^	330^c^ 310^c^	0.67 0.65	19^f^ 125^f^	/^g^ /^g^	703 683	Standalone device	[78]
	IrP_2_/NPC Pd_3_P/NPC PtP_2_/NPC	Ir = 5.3% Pd = 5.2% Pt = 4.8%	0.89^b^ 0.88^b^ 0.87^b^	350^c^ 360^c^ 370^c^	0.69 0.71 0.73	167^f^ 173^f^ 193^f^	/^d^ /^d^ /^d^	671 720 684	Standalone device	[79]
Oxide/ chalcogenide heterojunctions	RuCoO*_x_* Ru‐RuO_2_@NPC	/ Ru = 17.2%	0.85^b^ 0.79^b^	275^c^ 190^c^	0.66 0.63	37^c^ 79^c^	160^e^ /^e^	811.2 29	Standalone device full device	[80] [81]
Nanoparticle/ single‐atom hybrid Systems	Pt/NiO/Ni/CNT Ru‐Cl‐N SAC	Pt < 4% Ru ≈ 1.2%	0.94^b^ 0.90^b^	350^b^ 233^c^	0.64 0.56	125^b^ 12^c^	166.9^d^ 205.0^e^	708.2 804.2	Full device Full device	[32] [82]

Measurement conditions:

^a^ Δ*E* = *E* **_{OER, j = 10}_ −** *E* **_{ORR, 1/2}_** [74].

^b^ 0.1 m KOH.

^c^ 1 m KOH.

^d^ 6 m KOH.

^e^ 6 m KOH + 0.2 m Zn(Ac)_2_.

^f^ 6 m KOH + 0.2 m Zn(CH_3_COO)_2_.

^g^ 6 m KOH/6 m KOH + Zn(Ac)_2_.

Alloys and heterostructures leverage lattice strain, electronic synergy, and interfacial charge transfer to tune the d‐band center and surface adsorption. Sun et al. [75] synthesized fishbone‐like Pt‐rich PtCo/Ir‐rich IrCo ternary nanowires Figure 5a. With a Pt_62_Co_23_/Ir_15_ composition, they achieved an HER overpotential of 14 mV and an OER overpotential of 308 mV, with TOFs 10‐ and 4.5‐fold higher than Pt/C and Ir/C, respectively. A self‐powered Zn–air‐driven WS system reached 10 mA/cm^2^ at 1.53 V in acid and 23.3 times better than commercial Pt/C||Ir/C. However, this benchmark performance was demonstrated only in acidic media; the catalyst's durability and activity in alkaline or neutral conditions, which are more practical for ZABs, remain unexplored. Moreover, the ternary composition optimization required precise control, raising questions about reproducibility and scalability. Moving from morphology to electronic understanding, Zhang et al. [76] tuned Rh–Cu alloy ratios and showed that electron transfer from Cu to Rh induces asymmetric electron distribution (Figure 5b). Rh_6_Cu_1_ achieved a HER overpotential of 17 mV (acid) and an ORR half‑wave potential of 0.85 V. While this provides direct evidence of electronic synergy, the ORR activity (0.85 V) is modest compared to state‑of‑the‑art Pt‑based catalysts (typically >0.9 V). The study did not report OER performance, leaving tri‐functionality unconfirmed. Guided by interface engineering, Xiong et al. [77] prepared NF@Pt self‑supporting electrodes with ultralow Pt loading (0.71 wt.%). They revealed that the precious metal induces intrinsic activity in non‑precious components, enabling self‑powered WS driven by two Zn‑air cells in series. A key limitation is the lack of long‑term stability data under operating conditions. The induction mechanism was inferred from performance rather than directly probed by in situ characterization.

**FIGURE 5:**
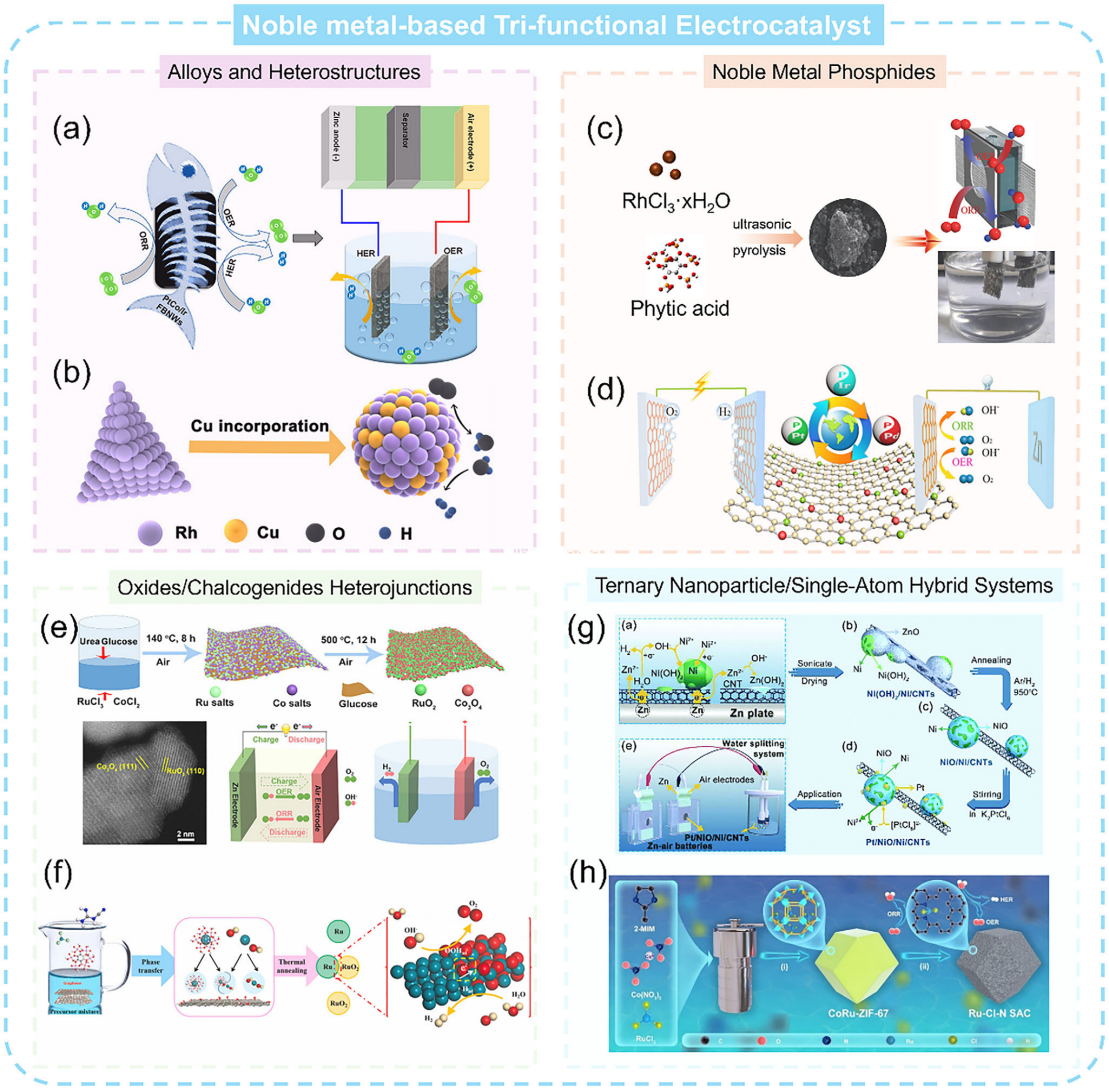
Noble metal‐based tri‐functional electrocatalyst. (a,b) Alloys and heterostructures. Reproduced with permission [75]. Copyright 2019, ACS. Reproduced with permission [76]. Copyright 2020, ACS. (c,d) Noble metal phosphides. Reproduced with permission [78]. Copyright 2018, Wiley. Reproduced with permission [79]. Copyright 2019, ACS. (e,f) Oxides/chalcogenides heterojunctions. Reproduced with permission [80]. Copyright 2021, ACS. Reproduced with permission [81]. Copyright 2021, Elsevier. (g,h) Ternary nanoparticle/single‐atom hybrid systems. Reproduced with permission [32]. Copyright 2020, RSC. Reproduced with permission [82]. Copyright 2022, Elsevier.

Noble metal phosphides combine precious metals with phosphorus, anchored on heteroatom‑doped carbon. Phosphorus atoms modulate the electronic structure and help construct tri‐functional sites. Qin et al. [78] synthesized Rh*_x_* P and RuP/NPC (0.4 wt.% Rh, 0.5 wt.% Ru) via phytic acid‑assisted pyrolysis (Figure 5c). Rh*_x_* P/NPC achieved an acid HER overpotential of 19 mV (TOF 0.45 H_2_/s, near Pt/C); RuP/NPC gave an OER overpotential of 310 mV (better than IrO_2_); both showed an ORR half‑wave potential of 0.89 V (40 mV positive shift vs. Pt/C). Despite these impressive metrics, the synthesis involves multiple steps and phytic acid, which may hinder scale‑up. More critically, the tri‐functional performance was measured in separate half‑cells; no device‑level demonstration (e.g., ZAB or water electrolyzer) was provided to validate real‑world applicability. Extending this concept, Qin et al. [79] synthesized IrP_2_/NPC, Pd_3_P/NPC, and PtP_2_/NPC (Figure 5d), confirming P‐metal and P─C bonds that stabilize nanoparticles. They elucidated surface restructuring of metal phosphides during OER. However, the restructuring mechanism was tracked ex situ or post‑mortem; operando evidence is still lacking. The generality of this strategy across different carbon supports was not examined.

Oxide/chalcogenide heterojunctions exploit interfacial charge coupling. Zhou et al. [80] prepared RuCoO*_x_* foam via a one‐step foaming process (Figure 5e), coupling RuO_2_ and Co_3_O_4_ in a 3D porous structure. Remarkable stability was reported: no degradation after 200 000 ORR cycles, and ZAB operated for 11 000 cycles (1833 h) at 10 mA/cm^2^ and 960 cycles at 100 mA/cm^2^, setting a record for tri‐functional catalysts. While stability is outstanding, the initial activity (OER overpotential not explicitly given in the highlighted text) may be moderate. The foam structure, though beneficial for mass transport, may suffer from mechanical fragility in flexible devices. Additionally, the synthesis used glucose as a foaming agent; the uniformity and reproducibility of the foam across batches need further validation. Wang et al. [81] constructed a Ru‐RuO_2_ Mott–Schottky‐type heterojunction on N, P‑codoped graphene (Figure 5f), showing activity across pH 0–14 and application in flexible solid‑state ZABs. The wide‑pH claim is based on half‑cell tests; flexible device performance under bending was not quantitatively reported.

Ternary nanoparticle/single‑atom hybrid systems maximize atom utilization. Bian et al. [32] used substrate‑enhanced electroless deposition to disperse ultrafine Pt nanoparticles (∼2 nm) on NiO/Ni/CNT Figure 5g. The catalyst achieved an ORR half‑wave potential of 0.942 V (mass activity 36.9× Pt/C), HER overpotential of 32.8 mV, and OER overpotential of 350 mV. A self‑powered WS system driven by two Zn‑air cells was demonstrated. The loading of Pt (not specified) likely remains non‑negligible. The NiO/Ni/CNT support contributed to activity, but the synergistic contribution of each component was not deconvoluted. Long‑term stability under dynamic load was not addressed. Chen et al. [82] synthesized a Ru single‑atom catalyst (Ru‑Cl‑N SAC) with bidentate Cl‑N coordination via ZIF‑67 templating (Figure 5h). Under alkaline conditions, HER overpotential reached 12 mV (TOF 2.29 H_2_/s, 9.5 times that of Pt/C), OER overpotential 233 mV, and ORR half‑wave potential 0.90 V, among the best reported. Despite the excellent half‑cell metrics, single‑atom catalysts often suffer from low metal loading (<1 wt.%), which limits volumetric activity. The stability of Ru single atoms under OER conditions, known to cause demetallation and agglomeration, was not rigorously evaluated over extended cycles. Furthermore, the self‑powered system was not actually assembled; the paper only claims potential.

In summary, the four strategies each have distinct emphases: alloys/heterostructures on d‑band and electron asymmetry; phosphides on P‑mediated regulation and surface restructuring; oxide/chalcogenide heterojunctions on Mott–Schottky effect and interfacial coupling; and nanoparticle/single‑atom systems on coordination engineering. At the mechanistic level, d‑band theory, coordination tuning, and in situ Raman tracking have advanced understanding, shifting the field from morphology‑based to electron‑oriented descriptions. At the application level, several studies have built Zn‐air‑driven self‑powered WS systems and extended to flexible wearables.

However, persistent challenges remain: (1) High cost and scarcity are fundamental barriers and reducing precious metal usage without compromising performance requires further breakthroughs, especially in single‑atom catalysts where ultra‑low loading often leads to low absolute activity; (2) A unified model for tri‐functional active sites is lacking and the atomic‑scale requirements for HER, OER, and ORR are often contradictory; most studies optimize one or two reactions at the expense of the third; (3) Interface evolution and activity decay during long‑term cycling remain poorly understood and operando characterization is still underutilized. Future research should integrate in situ/operando techniques with AI‑assisted high‑throughput screening to transition from precious metal dependence to rational design. Concurrently, interface engineering principles developed for precious metals should be adapted to non‑precious metal systems, ultimately enabling high‑performance, low‑cost, and long‑lasting tri‐functional catalysts.

### 3.3 Metal‐Based Tri‐Functional Electrocatalyst

Metal‐based tri‐functional (ORR/OER/HER) electrocatalysts are key to practical ZABs and water electrolysis. Recent advances broadly fall into three categories: monometallic, bimetallic, and alloy‑based systems (Table 3 summarizes representative noble metal‐based tri‐functional electrocatalysts), each with distinct activity origins, but also common limitations in site identification, reaction balance, and operational stability.

**TABLE 3:** Summary of some metal‐based tri‐functional electrocatalysts.

Classification	Catalyst	*E*_1/2_ORR (V)	*η*_10_OER (mV)	Δ*E* (V)^a^	*η*_10_HER (mV)	Power density (mW/cm^2^)	Specific capacity (mAh/g)	Device validation	Refs.
Commercial benchmarks	Pt/C (20 wt.%)	0.88^b^	/	/	25^c^	/	/	Commercial	[72]
	IrO_2_	/	360^c^	/	/	/	/	Commercial	[73]
Monometallic	Fe‐N‐C/FeP/NPSC	0.90^b^	370^c^	0.70	198^c^	216.88^d^	712.9	Full device	[83]
	FeP/IF	/	397@100^c^	/	242@100^c^	/^e^	/	Full device	[84]
	CoP@CNS	0.76^c^	286@50^c^	/	91^c^	110^d^	/	Standalone device	[85]
	GF/Co@NCNTs	0.85^b^	260^c^	0.64	195^c^	78.33^d^	718.05	Full device	[25]
	Ni@N‐HCGHF	0.87^b^	260^c^	0.62	95^c^	117.1^d^	706	Full device	[87]
	NiMOF‐Ni_2_P/IF	/	240^c^	/	86^c^	/	/	Standalone device ^f^	[60]
Bimetallic	CoFe‐LDH@NPC	0.79^b^	280^c^	0.72	100^c^	58.39^g^	635.93	Standalone device	[88]
	CoNC_HS_/Cu_NWs_	/^b^	289@20^c^	/	163@20^c^	/^d^	/	Standalone device	[38]
	CoMoO_4_/Co_3_O_4_@CC	/^c^	250^c^	/	/	120.3^d^	654	Standalone device	[89]
	Co_9_S_8_‐CoMo_2_S_4_/NSC	0.86^b^	280^c^	0.65	89^c^	112.82^d^	804	Full device	[42]
	V_O_‐CoWO_4_	/	260^c^	/	/	152.2^h^	573.2	Standalone device	[90]
Alloy	NCMP	0.76^b^	218@50^c^	/	100^c^	148^f^	740.3	Standalone device	[18]
	Mo‐NiFe_2_O_4_/IF	/	275.4@100^c^	/	311.7@100^c^	106^e^	/	Full device	[91]
	Zn‐FeCo@CNF‐900	0.84^b^	269^c^	0.66	169^c^	195^d^	735.6	Full device	[19]
	FNCO‐600	0.76^b^	230^c^	0.70	211^c^	21.8^d^	973	Full device	[33]

Measurement conditions:

^a^ Δ*E* = *E* **_{OER, j = 10}_ −** *E* **_{ORR, 1/2}_** [74].

^b^ 0.1 m KOH.

^c^ 1 m KOH.

^d^ 6 m KOH + 0.2 m Zn(Ac)_2_.

^e^ 6 m KOH + 0.02 m Zn(Ac)_2_.

^f^ 6 m KOH.

^g^ PVA‐KOH. 6 m KOH + ZnO.

^h^ 6 m KOH + 0.2 m Zn(Ac)_2_ + 0.5 m K_2_WO_4_.

Recent progress in monometallic catalysts can be broadly classified into three categories: Fe‑based, Co‑based, and Ni‑based catalysts. Their achievements and remaining challenges are discussed below. In iron‐based systems, Fe‑N‑C materials have emerged as a research frontier due to their cost advantage and catalytic potential. Li et al. [83] proposed a site‐coupling strategy using a salt‐assisted templating method to integrate the ORR‐active FeN*_x_* center and the HER‐active Fe*_x_* P phase into an N, P, S‑doped carbon framework, achieving tri‐functional catalysis through synergy Figure 6a. They further validated the system from materials to devices: a liquid ZAB showed high power density and long cycling stability; a quasi‑solid‑state flexible battery powered an LED even under bending, and series connection enabled full WS. However, three limitations remain: (1) the synergistic mechanism among N, P, S dopants was not quantitatively resolved—which element dominates is unclear; (2) the Fe*_x_* P phase is prone to surface reconstruction under OER conditions, and long‑term stability was not adequately assessed; (3) the quasi‑solid‑state device lacked systematic optimization of the electrode‐electrolyte interface, and performance decay after repeated bending cycles was not reported. Shifting to electrode architecture, Wang et al. [84] improved the structural stability and mass transport of FeP by designing a self‑supporting electrode Figure 6b, and further assembled a prototype storage‑to‑conversion self‑sustaining hydrogen system driven by ZABs in series. The main drawbacks are that the preparation of the self‑supporting electrode (using Ni foam) makes precise control of FeP loading and distribution difficult; the energy efficiency of the series system was not reported, only a feasibility demonstration; and phosphorus loss from FeP under long‑term operation was not tracked.

**FIGURE 6:**
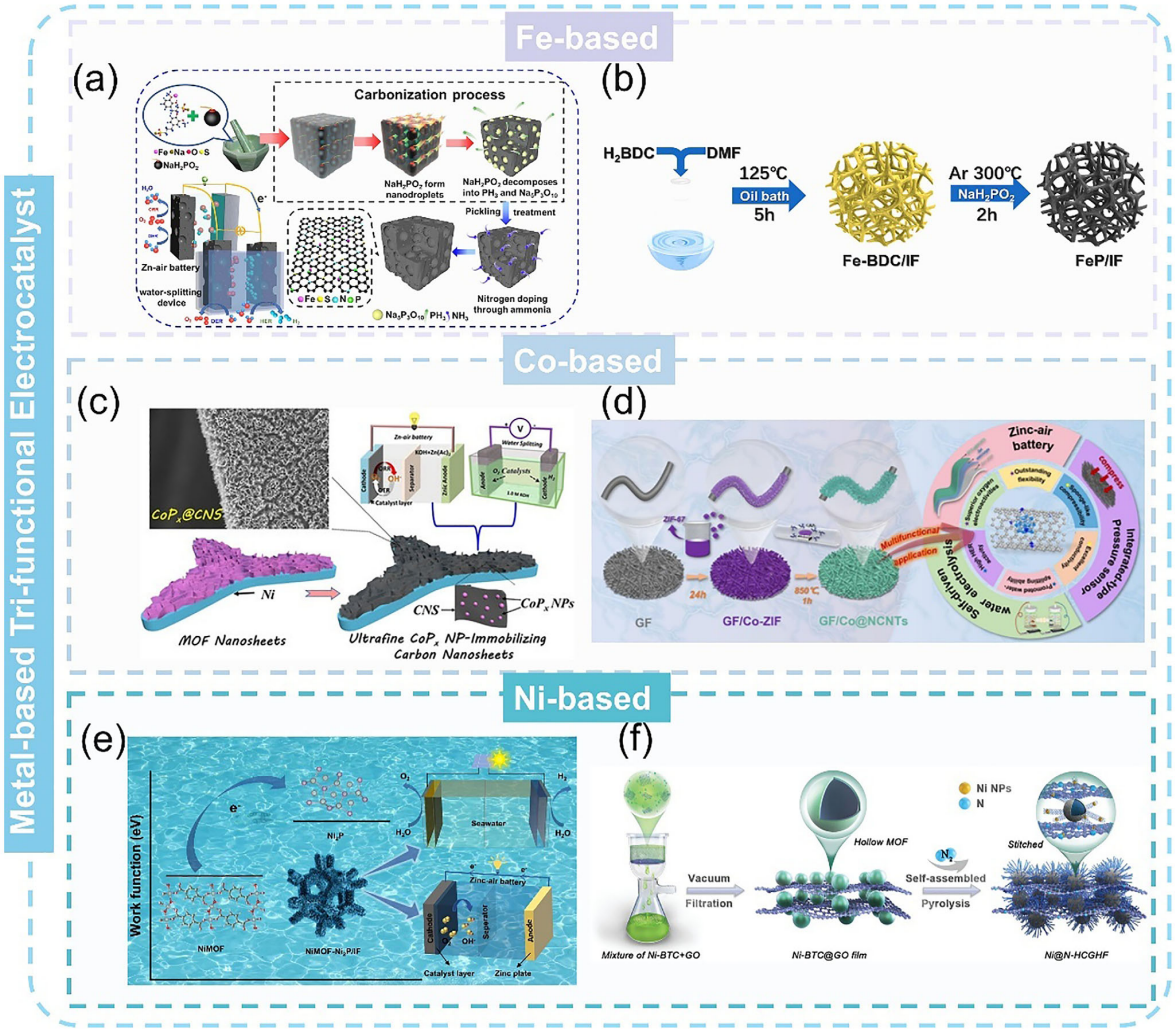
Metallic tri‐functional electrocatalyst. (a,b) Iron‐based systems. Reproduced with permission [83]. Copyright 2021, Elsevier. Reproduced with permission [84]. Copyright 2026, Elsevier. (c,d) Cobalt‐based systems. Reproduced with permission [85]. Copyright 2020, Wiley. Reproduced with permission [25]. Copyright 2025, Elsevier. (e,f) Nickel‐based systems. Reproduced with permission [87]. Copyright 2021, Wiley. Reproduced with permission [60]. Copyright 2021, Elsevier.

In cobalt‐based systems, Co‑based materials offer tunable electronic structures, diverse active sites, and good conductivity. Early breakthroughs focused on electrode structure innovation. Hou et al. [85] used 2D Co‐MOF nanosheets as precursors to construct a 3D porous self‐supporting electrode (CoPx@CNS) on Ni foam Figure 6c, demonstrating a complete chain from MOF design to ZAB and WS devices. The limitations include low yield and high cost of the MOF precursor, hindering scale‑up; pore blocking by gas bubbles under dynamic conditions was not analyzed; and the phase reconstruction of cobalt species under OER was not investigated. With the electrode architecture established, research shifted to mechanistic understanding. Liang et al. [25] systematically revealed the coordination environment of Co‑N*_x_*‑C active sites and their electronic synergy in a graphite‑felt‑supported Co@NCNT system Figure 6d. Nevertheless, this study relied mainly on ex situ XPS and DFT calculations; operando characterization to track the dynamic evolution of Co sites was missing. Moreover, the ORR half‑wave potential was not clearly specified, and HER/OER tests were performed in different electrolytes, not under unified conditions.

In nickel‐based systems, Ni‑based materials are attractive due to variable valence states, abundant active phases, and excellent stability [86]. Yan et al. [87] constructed a self‑supporting 3D hetero‐structured film using a MOF‑assisted strategy and claimed atomic‑scale understanding of its high performance Figure 6e. However, the proposed atomic‑scale mechanism was based solely on DFT and static characterization; no in situ Raman or XAS evidence was provided to track Ni valence changes during reactions. The mechanical robustness of the 3D structure is insufficient for flexible device applications. Building on this, He et al. [60] in Figure 6f established a stable carbon‑encapsulated metal compound structure, preparing ready‑to‑use self‑supporting NPO/NixPy@NF‐HPCs electrodes, and bridging porous carbon preparation, in situ composite growth, and device integration. A critical issue is that carbon encapsulation, while protecting the metal, may also hinder active site exposure to the electrolyte; the activity difference before and after encapsulation was not compared. Furthermore, bubble detachment behavior under high current densities was not evaluated.

Although single‑metal tri‐functional catalysts have advanced, their further development faces inherent bottlenecks. Fe‑based materials: Fe‐N‑C sites excel in ORR but suffer from oxidative dissolution under high‑potential OER conditions, compromising stability. Moreover, the electronic structure of single‑iron species cannot simultaneously optimize the adsorption energies of intermediates across ORR, OER, and HER. Co‑based materials: They offer good conductivity and tunable electronic states, but cobalt is relatively scarce and costly, and its intrinsic activity across a wide pH range still needs improvement. Ni‑based materials: They exhibit outstanding stability, yet their ORR activity is typically inferior to that of Fe‑ and Co‑based systems, often requiring complex nano structuring to compensate for insufficient active site density. A common challenge across all single‑metal systems is the trade‑off among activity, stability, and cost. Moreover, a single active center cannot adequately serve three distinct reactions. These limitations have driven interest in bimetallic catalysts, where a second metal introduces electronic synergy and coordination modulation, offering a pathway to break through the performance ceiling of single‑metal materials and achieve balanced optimization of activity, stability, and cost.

In bimetallic tri‐functional electrocatalyst systems, morphology engineering optimizes mass transport, exposes active sites, and enhances structural stability. Proper micro‑/macro‑structure design creates an ideal environment for bimetallic sites, maximizing intrinsic activity and long‑term durability. Li et al. [88] prepared layered N‑doped porous carbon nanosheets as a conductive scaffold, then grew CoFe layered double hydroxide (LDH) nanosheets on the surface, forming a CoFe‑LDH@NPC hierarchical structure Figure 7a. This design suppressed LDH stacking, increased site accessibility, and enabled high tri‐functional activity. Assembled WS and flexible ZABs showed good performance. However, the study did not quantify how much the hierarchical structure improved mass transport compared to a non‑hierarchical control. The long‑term stability of the LDH phase under OER conditions was not monitored by operando techniques. Moreover, the flexibility of the device was only qualitatively demonstrated; no bending‑cycle stability data were provided. Furthermore, He et al. [38] anchored Co, Cu, N‑doped carbon dodecahedra (CoCu‐N‐C) onto copper nanowire chains on a flexible copper mesh, forming a bead‑chain interlocked structure (CoNCHS/CuNws) Figure 7b. This binder‑free, self‑supporting electrode offered open pore channels and mechanical robustness. A key limitation is that the copper nanowires are prone to oxidation in alkaline electrolytes were not addressed. The reported performance was measured in half‑cells; device‑level testing (e.g., in an actual ZAB under bending) was not performed. Additionally, the role of the CoCu‑N‐C dodecahedra versus the CuNW substrate in the overall activity was not deconvoluted.

**FIGURE 7:**
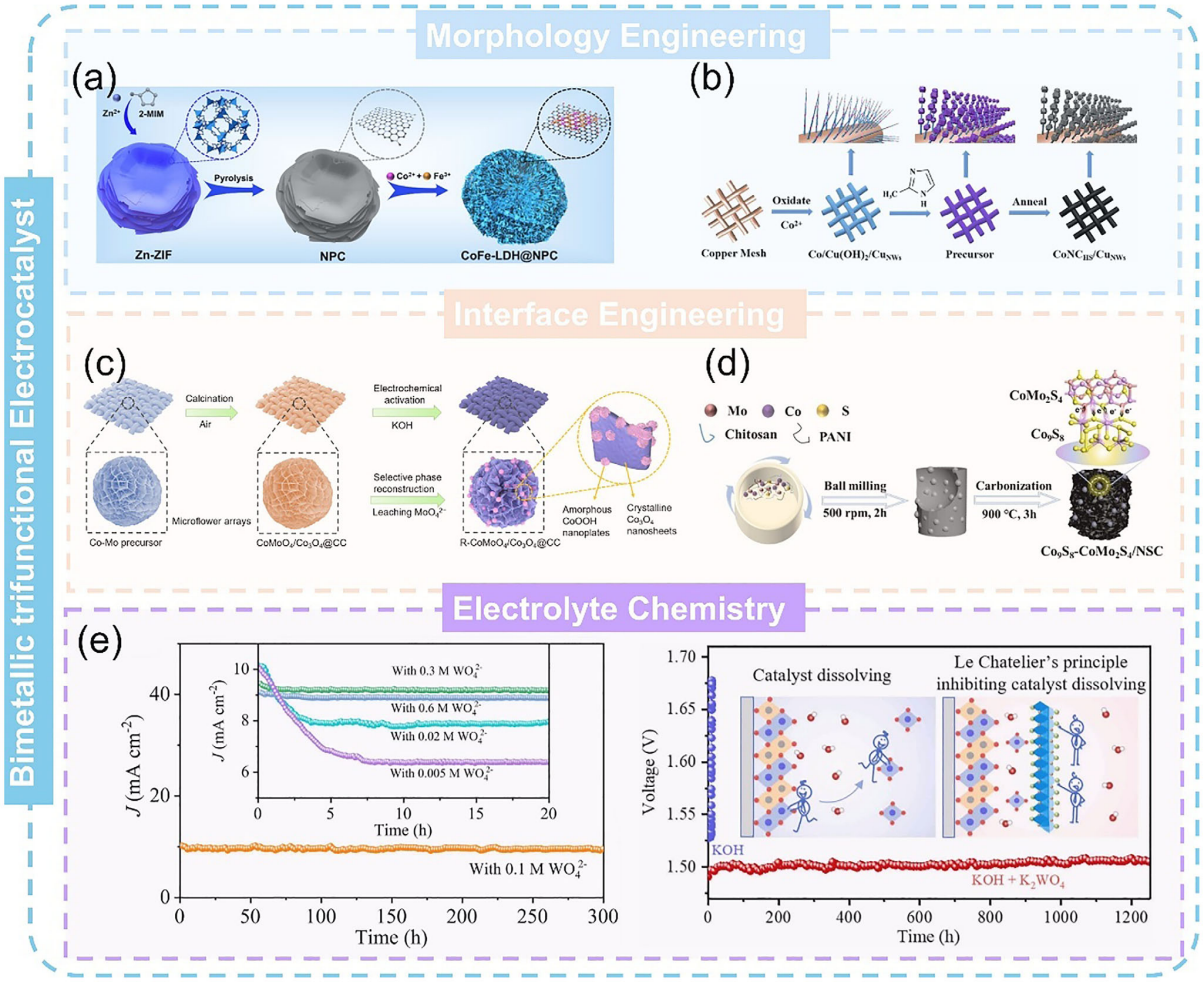
Bimetallic tri‐functional electrocatalyst. (a,b) Morphology engineering. Reproduced with permission [88]. Copyright 2023, ACS. Reproduced with permission [38]. Copyright 2026, Elsevier. (c,d) Interface engineering. Reproduced with permission [89]. Copyright 2020, Elsevier. Reproduced with permission [42]. Copyright 2025, CSC. (e) Electrolyte chemistry. Reproduced with permission [90]. Copyright 2021, Elsevier.

As morphology engineering matures, focus shifts to interface engineering for intrinsic activity regulation. Two main approaches have emerged: dynamically reconfigurable interfaces and static heterojunctions. Zhu et al. [89] used selective dissolution or restructuring of one component under electrochemical conditions to trigger a controlled phase transition in another, dynamically constructing an amorphous/crystalline heterojunction in situ Figure 7c. The limitation lies in the lack of generalizability: this strategy depends on specific material pairs that undergo such transformations. The study did not provide a predictive criterion for which systems would work. Moreover, the dynamic interface was characterized ex situ after reconstruction, not tracked in real time during operation. Wang et al. [42] directly coupled two crystalline phases, Co_9_S_8_ (ORR/OER) and CoMo_2_S_4_ (HER), via a one‐pot ball‐milling and pyrolysis process Figure 7d. Strong interfacial electronic synergy optimized intermediate adsorption, achieving 1 + 1 > 2 performance. However, the ball‑milling process may introduce inhomogeneity; the interface density and distribution were not quantified. The stability of the heterojunction under long‑term cycling was not evaluated. Furthermore, the synthesis required high‑temperature pyrolysis, limiting scalability. Beyond material design, regulating the electrolyte environment represents a paradigm shift. Zhu et al. [90] use a V_O_‐CoWO_4_ catalyst rich in oxygen vacancies Figure 7e, where high OER activity caused selective dissolution of W species. By adding WO_4_^2^^−^ ions to the electrolyte, they applied Le Chatelier's principle to reversible dissolution, extending device lifetime without sacrificing activity. This elegant approach has its own constraints: the added ions may alter electrolyte pH or ionic strength, affecting other reactions (e.g., HER or ORR).

The study focused only on OER; the tri‐functionality of the system under the same electrolyte modulation was not examined. Additionally, this strategy is specific to dissolution‑prone elements; its applicability to other degradation modes (e.g., phase transformation, agglomeration) remains unproven. In summary, bimetallic catalysts enhance tri‐functional performance through multi‑level synergy of morphology, interfaces, and electrolytes. However, most structures rely on heterojunctions or composite phases with relatively discrete interfaces. To achieve more uniform and robust electronic control, research has naturally moved toward alloy tri‐functional catalysts.

Alloy tri‐functional electrocatalysts enable atomic‑level electronic modulation, offering an ideal platform for high intrinsic activity, stability, and conductivity. Current research proceeds along three parallel directions: high‑loading electrode engineering for industrialization, theory‑guided rational design, and flexible integrated devices for practical applications. In high‐loading electrode engineering, Khodayar et al. [18] addressed the industrial bottlenecks of high loading by synthesizing a ternary metal phosphide with an interconnected nanostructure, forming a binder‑free integrated electrode Figure 8a. The morphology ensures good ion/electron pathways and site accessibility even under high loading. However, the study did not report the actual loading mass or compare performance at different loadings. The long‑term mechanical stability of the interconnected structure under gas evolution (e.g., OER) was not evaluated. Moreover, the synthesis complexity and scalability for industrial production remain unaddressed. In theory‑guided rational design, Chi et al. [19] used DFT calculations to pre‑reveal electronic structure modulation by doping/alloying Figure 8b, then capitalized on the low boiling point of Zn to create a micro‑/mesoporous carbon framework, which in turn immobilized the alloy nanoparticles. This increased surface area, promoted mass transport, and protected active sites. A key limitation is that DFT predictions were made for ideal surface models, yet the actual alloy nanoparticles have multiple facets and defects not captured by the calculations. The study did not validate whether the predicted doping sites matched the experimental ones via atomic‑scale characterization. Furthermore, the DFT‑to‑synthesis loop was one‑way; no iterative feedback from experiment to refine the model was performed. In flexible integrated devices, Zheng et al. [91] grew Mo‐doped NiFe_2_O_4_ nanoflowers on iron foam Figure 8c, enhancing OER/HER at high current densities. Meanwhile, Kumar et al. [33] prepared a self‑supporting, foam‑like interconnected porous FeNiCoO_4_ catalyst via citrate pyrolysis Figure 8d. The carbon‑free 3D morphology provides abundant active sites and rapid mass transport, enabling good OER/HER/ORR performance, and was successfully applied in solid‑state and liquid ZABs for flexible electronics. Both studies share common weaknesses: the flexible device performance under repeated bending cycles was only qualitatively shown, without quantitative retention data. The self‑supporting electrodes were tested in half‑cells under mild conditions; their performance under practical stressors (e.g., high current density, mechanical deformation, and long‑term cycling simultaneously) was not reported. Additionally, the FeNiCoO_4_ catalyst contains no carbon, but the citrate pyrolysis may leave residual amorphous carbon that is not characterized. The leaching of transition metals (Ni, Co, Fe) under OER conditions was not examined. In summary, alloy tri‐functional catalysts are moving from component exploration toward multidimensional, application‑driven development. However, high‑loading electrodes still face scalability questions; theory‑guided design often lacks experimental validation of predicted atomic structures; and flexible devices need rigorous dynamic stability testing. Future work should close the feedback loop between computation and experiment, establish standardized high‑loading evaluation protocols, and incorporate real‑world mechanical and electrochemical stress tests for flexible systems.

**FIGURE 8:**
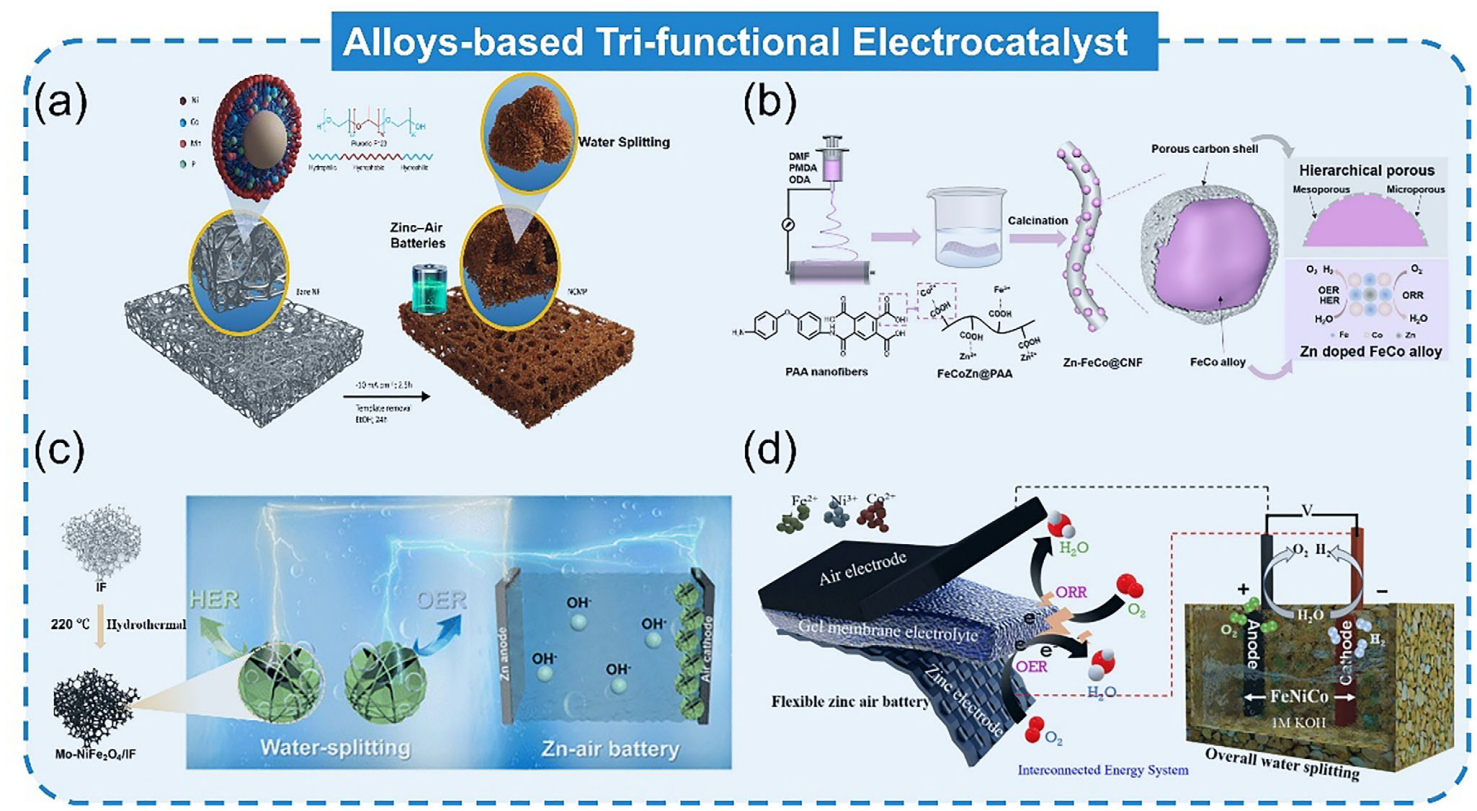
Alloy tri‐functional electrocatalyst. (a) Reproduced with permission [18]. Copyright 2024, RSC. (b) Reproduced with permission [19]. Copyright 2025, Elsevier. (c) Reproduced with permission [91]. Copyright 2025, RSC. (d) Reproduced with permission [33]. Copyright 2025, ACS.

Research on metal‑supported tri‐functional electrocatalysts has evolved into a hierarchical, multi‑dimensional system. The field is shifting from performance‑driven rapid growth toward rational design guided by mechanistic understanding and practical needs. However, several key issues remain unresolved. First, the true active phase under OER conditions often deviates from the as‑synthesized one because sulfides, phosphides, and nitrides may partially transform into oxyhydroxides. Second, deconvoluting the contribution of each component in multi‑element catalysts is challenging. Third, most stability tests are still conducted under mild laboratory conditions, which poorly reflect long‑term operation in practical ZABs or electrolyzers. Addressing these gaps is essential for advancing metal‑based catalysts toward sustainable energy applications.

### 3.4 Heterogeneous Tri‐Functional Electrocatalyst

Heterojunction engineering is a core strategy for non‑precious metal tri‐functional electrocatalysts. A heterojunction is defined as an interface between two distinct material phases with different work functions or band structures, which generates a built‑in electric field that optimizes intermediate adsorption, promotes charge transfer, and stabilizes active sites [86, 87, 88, 89, 90, 91, 92]. It uses built‑in electric fields at material interfaces to drive charge redistribution, thereby optimizing multi‑step reaction kinetics. The goal is not merely combining components but achieving synergistic performance beyond simple summation. Based on interface formation mechanisms, heterojunctions fall into four types: metal‑semiconductor heterojunctions, p‑n heterojunctions, structure‑defined heterojunctions, and composition/doping‑mediated heterojunctions (Table 4 summarizes representative heterogeneous tri‐functional electrocatalysts). Their common aim is to overcome the intrinsic limitations of single materials and enable efficient ORR, OER, and HER synergy.

**TABLE 4:** Summary of some heterostructured tri‐functional electrocatalysts.

Classification	Catalyst	*E*_1/2_ORR (V)	*η*_10_OER (mV)	Δ*E* (V)^a^	*η*_10_HER (mV)	Power density (mW/cm^2^)	Specific capacity (mAh/g)	Device validation	Refs.
Commercial benchmarks	Pt/C (20 wt.%)	0.88^b^	/	/	25^c^	/	/	Commercial	[72]
	IrO_2_	/	360^c^	/	/	/	/	Commercial	[73]
Heterostructures	Co_2_P/Co@N‐CNT/NPG	0.91^b^	390^c^	0.71	98^c^	745^d^	781@10	Full device	[92]
	FeNi/FeNiO‐NCS	0.86^b^	300^c^	0.67	210^c^	156^d^	855	Full device	[93]
	LaCoO_3_/NiFe LDH	/	260^c^	/	/	/^d^	/	Standalone device	[65]
	Co_9_S_8_/CuCo_2_S_4_	0.79^b^	264^c^	0.70	191^c^	89.24^d^	706.6	Full device	[94]
	CoFe‐NiFe/NC	0.85^b^	185^c^	0.57	66^c^	155^d^	809@10	Full device	[95]
	Fe,CoZn_9+9_‐NO/WC	0.89^b^	290^c^	0.63	84^c^	190^d^	814	Standalone device	[21]
	Co‐S‐INF	0.63^b^	248@100^b^	/	289@100^c^	160.5^d^	778.9	Standalone device	[96]

Measurement conditions:

^a^ Δ*E* = *E* **_{OER, j = 10}_ −** *E* **_{ORR, 1/2}_** [74].

^b^ 0.1 m KOH.

^c^ 1 m KOH.

^d^ 6 m KOH + 0.2 m Zn(Ac)_2_.

Metal‐semiconductor heterojunctions rely on work function differences to form Mott–Schottky junctions, inducing spontaneous electron transfer from metal to semiconductor. Two synthesis approaches dominate. One‑pot precursor integration is efficient and scalable. Ngo et al. [92] mixed graphene oxide, cobalt salt, dicyandiamide, and an organic phosphorus source, then used single‑step pyrolysis to simultaneously form Co_2_P/Co heterojunctions, a N, P‑doped carbon network, and encapsulated active particles Figure 9a. This simplified process yields high activity, conductivity, and stability. However, the one‑pot method offers limited control over heterojunction density and interface quality; different precursors may pyrolyze at different rates, leading to batch‑to‑batch variation. The study did not quantify the fraction of Co_2_P versus Co phases, nor assess whether unreacted precursors left detrimental residues. Stepwise precision assembly provides atomic‑scale control. Xin et al. [93] prepared negatively charged Fe, N‑doped carbon nanosheets via exfoliation‑intercalation‑assembly, then assembled them with positively charged FeNi LDH layers. Controlled pyrolysis yielded metallic FeNi_3_ and semiconducting FeNiO nanoparticles tightly coupled at the atomic scale and anchored on a N‑doped carbon framework Figure 9b. The three‑step process is complex and time‑consuming, limiting scalability. The study did not demonstrate whether the tight atomic contact survives under harsh OER conditions, where lattice expansion or gas evolution might disrupt the interface. Moreover, the specific contribution of the heterojunction versus the carbon framework was not deconvoluted. P‐n heterojunctions represent a shift from macroscopic charge regulation to band structure engineering. Cui et al. [65] combined p‐type LaCoO_3_ with the n‐type NiFe LDH to form a Type‐II p‐n junction with efficient charge separation, applied to photo‐assisted electrocatalytic OER Figure 9c. The work focuses solely on OER; trifunctionality (HER/ORR) was not demonstrated. The photo‑assisted enhancement under real sunlight (rather than a solar simulator) and the long‑term stability of the perovskite‑LDH interface under illumination were not reported. Additionally, the energy efficiency of the photo‑assisted system was not benchmarked against conventional electrocatalysis. Structure‑defined heterojunctions move toward rational design of specific spatial configurations. Zhang et al. [94] used a self‑sacrificing template to grow vertically aligned hollow tubular heterojunctions on flexible carbon fabric Figure 9d. Template‑guided growth simultaneously defined orientation, dimensions, porosity, and interfaces. A key limitation is that the hollow tubular structure, while beneficial for mass transport, is mechanically fragile; flexibility under repeated bending was not quantified. The template removal step may leave residues that block active sites. Moreover, the reported tri‐functional performance was measured in separate half‐cells; device‐level testing (e.g., in a flexible ZAB under deformation) was missing. Yang et al. [95] demonstrated multi‐scale integration by embedding 0D biphasic nanoalloys into 2D N‐doped carbon nanosheets, assembled on a 3D Ni foam scaffold nickel foam scaffold Figure 9e. This self‑template‑assisted ion adsorption‑pyrolysis strategy yielded ultra‑high site density, a robust conductive network, and abundant interfaces. The synthesis involves multiple steps and precise control of ion adsorption, which is difficult to scale. The study did not address the long‑term stability of the 3D scaffold under high‑current‑density OER, where Ni foam is known to corrode in alkaline media. The contribution of the Ni foam substrate to the measured activity was not subtracted or separately evaluated. Composition/doping‑mediated heterojunctions use heteroatom doping or compositional gradients to induce built‑in electric fields within a single phase, avoiding lattice mismatch. Xue et al. [21] co‑doped Fe, Co, N, and O into a 3D WC matrix via ZIF templating, yielding carbon nanotubes with electronic structure gradients Figure 9f. The study did not quantify the distribution of dopants across the material; it is unclear whether the gradient is continuous or merely statistical. The role of residual carbon from ZIF pyrolysis was not separated from the WC‑derived activity. Most importantly, the tri‐functional performance was reported only in alkaline media; activity in acidic or neutral conditions, relevant for certain electrolyzers, was not explored. Dang et al. [96] used electrodeposition‑hydrothermal synthesis to fabricate a Co_3_S_4_/(Fe,Ni)_9_S_8_ nanoflower heterojunction on FeNi foam Figure 9g. Performance was attributed to the well‑defined interface. The two‑step method introduces complexity; the interface density was not quantified, and the possibility of elemental interdiffusion during hydrothermal treatment was not examined. The FeNi foam substrate may actively participate in OER, but its contribution was not deconvoluted. Long‑term cycling at practical current densities was not reported. In summary, the evolution from metal‐semiconductor heterojunctions, p‐n heterojunctions, and structure‐defined heterojunctions reflects a deeper shift in electronic regulation. However, several common gaps persist: (1) most studies rely on half‑cell testing without device‑level validation; (2) synthesis complexity and scalability are rarely addressed; (3) the real active phase under operation (especially for sulfides and phosphides) is often different from the as‑prepared one; (4) the contribution of the substrate or carbon matrix is seldom deconvoluted. Future work should prioritize operando characterization of interface dynamics, standardized device‑level testing, and scalable synthesis routes that retain atomic interface control.

**FIGURE 9:**
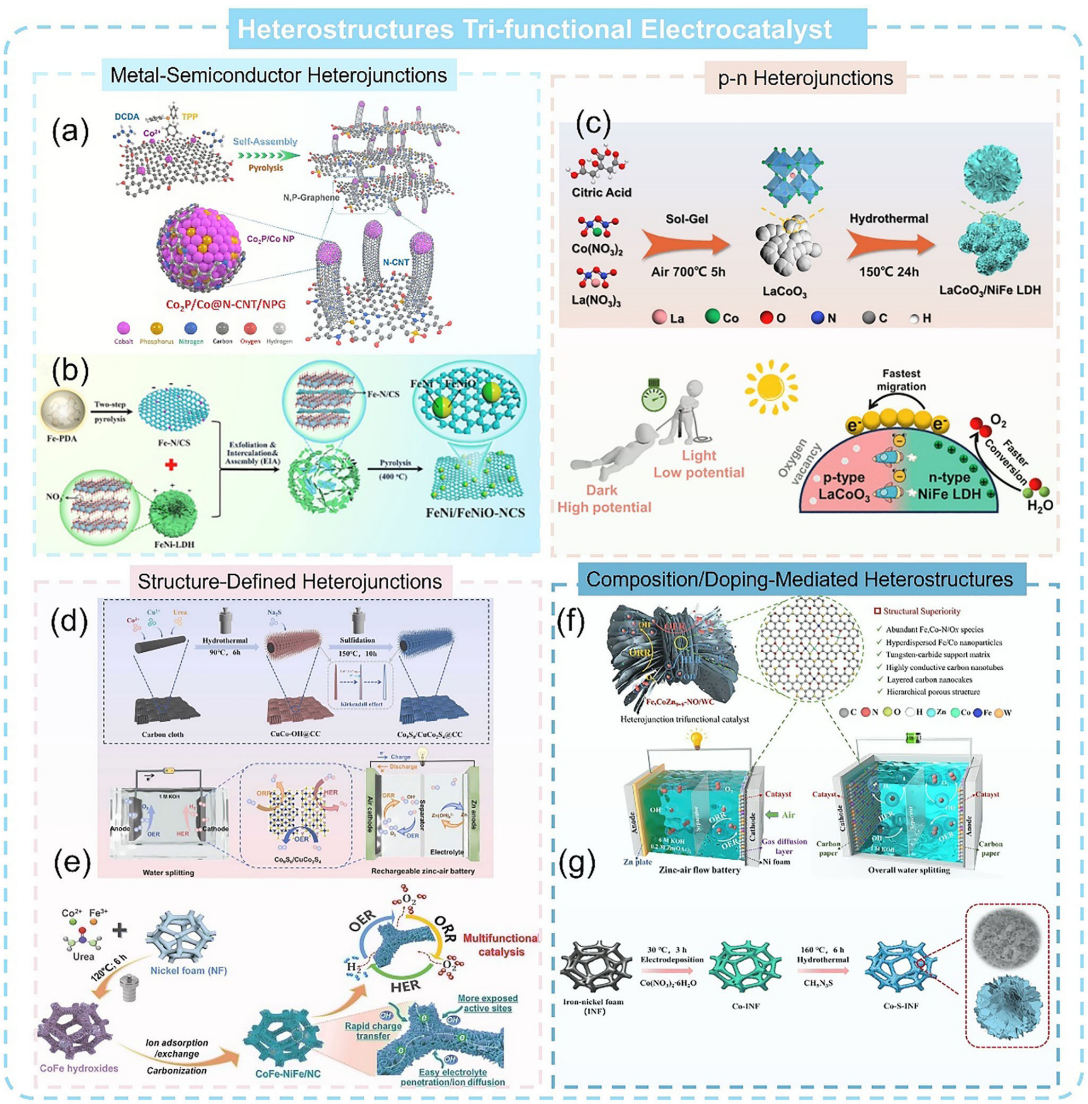
Heterostructures tri‐functional electrocatalyst. (a,b) Metal semiconductor heterojunctions, and composition/doping‐mediated heterojunctions. Reproduced with permission [92]. Copyright 2023, RSC. Reproduced with permission [93]. Copyright 2025, Wiley. (c) p‐n heterojunctions. Reproduced with permission [65]. Copyright 2024, ACS. (d,e) Structure‐defined heterojunctions. Reproduced with permission [94]. Copyright 2025, Elsevier. Reproduced with permission [95]. Copyright 2024, Wiley. (f,g) Structure‐defined heterojunctions. Reproduced with permission [21]. Copyright 2023, Elsevier. Reproduced with permission [96]. Copyright 2021, Elsevier.

### 3.5 Single‐Atom Tri‐Functional Electrocatalyst

Single‑atom catalysts (SACs) maximize atomic utilization and offer tunable coordination microenvironments, making them attractive for tri‐functional electrocatalysis. The core challenge is stabilizing atomically dispersed metal centers while precisely controlling the central metal, ligands, and spatial configuration to balance adsorption energies for ORR, OER, and HER. The field has moved from constructing isolated single sites to building multi‑metal synergistic systems. Table 5 summarizes recent single‑atom tri‐functional catalysts.

**TABLE 5:** Summary of some single‐atom trifunctional electrocatalysts.

Classification	Catalyst	*E*_1/2_ORR (V)	*η*_10_OER (mV)	Δ*E* (V)^a^	η_10_HER (mV)	Power density (mW/cm^2^)	Specific capacity (mAh/g)	Device validation	Refs.
Commercial benchmarks	Pt/C (20 wt.%)	0.88^b^	/	/	25^c^	/	/	Commercial	[72]
	IrO_2_	/	360^c^	/	/	/	/	Commercial	[73]
Single atom	CoN_4_‐C‐NiN_4_‐C‐FeN_4_ TSAs	0.88^b^	393^b^	0.74	94^b^	104^d^	728.3	Standalone device	[97]
	Ru‐Cl‐N SAC	0.90^b^	233^c^	0.56	12^c^	205^e^	804.26	Full device	[82]
	Co_2_P/Co‐N‐C	0.89^b^	330^c^	0.67	190^c^	91^f^	700	Full device	[98]

Measurement conditions:

^a^ Δ*E* = *E* **_{OER, j = 10}_ −** *E* **_{ORR, 1/2}_** [74].

^b^ 0.1 m KOH.

^c^ 1 m KOH.

^d^ 0.2 m ZnCl_2 _+ 6 m KOH.

^e^ 6 m KOH + 0.2 m Zn(OAc)_2_.

^f^ 6 m KOH.

In a ternary single‑atom system, Yang et al. [97] overcame the limitation of single‑metal sites by synthesizing a ternary SAC (CoN_4_‐C‐NiN_4_‐C‐FeN_4_) using a metal‐encapsulation‐isolation‐coating strategy Figure 10a. DFT suggested long‑range electronic interactions among Fe, Ni, and Co sites: FeN_4_ dominates ORR, NiN_4_ dominates OER, and CoN_4_ dominates HER, with neighboring sites modulating each other. However, several issues remain: (1) the actual metal loadings of Fe, Ni, and Co were not independently quantified; (2) long‑range electronic interactions were inferred solely from DFT without direct experimental evidence (e.g., from XPS or XANES); (3) the stability of three different single‑atom species under prolonged OER cycling was not assessed; (4) the synthesis complexity raises concerns about batch‑to‑batch reproducibility. In local coordination engineering, Chen et al. [82] used synchrotron XAS and DFT to identify a Ru‑Cl‑N SAC with a RuCl_2_N_2_ coordination, rather than the typical RuN_4_ Figure 10b. The highly electronegative Cl atoms altered the Ru electronic density of states, optimizing adsorption free energies for HER, OER, and ORR, thereby surpassing commercial benchmarks. The limitations are significant: (1) Cl ligands are prone to detachment under oxidative OER conditions, but the study did not monitor Cl retention post‑cycling; (2) the catalyst was tested only in alkaline media; performance in acid or neutral conditions is unknown; (3) the Ru loading was not reported, and single‑atom catalysts typically suffer from low absolute activity due to <1 wt.% loading; (4) no device‑level demonstration (e.g., in a ZAB or water electrolyzer) was provided. In a single‐atom and nanoparticle hybrid system, Liu et al. [98] moved beyond conventional single‐site design by constructing a composite of Co–N*_x_* species and hollow Co_2_P nanoparticles Figure 10c. They proposed that electron transfer from Co_2_P to the Co–N*_x_* sites at the interface optimizes the d‑band structure, enhancing oxygen intermediate adsorption/transformation. The unresolved questions include: (1) the electron transfer direction was deduced from DFT, but no experimental charge density mapping (e.g., by EELS or XPS) was shown; (2) the actual interface between single atoms and nanoparticles is poorly defined; Co_2_P is known to reconstruct into CoOOH under OER conditions, so the claimed stable interface may be transient; (3) the study did not separate the contributions of Co–N*_x_* versus Co_2_P via control experiments; (4) tri‐functional performance was reported only in half‑cells, and long‑term cycling stability was not examined under device‑relevant conditions.

**FIGURE 10:**
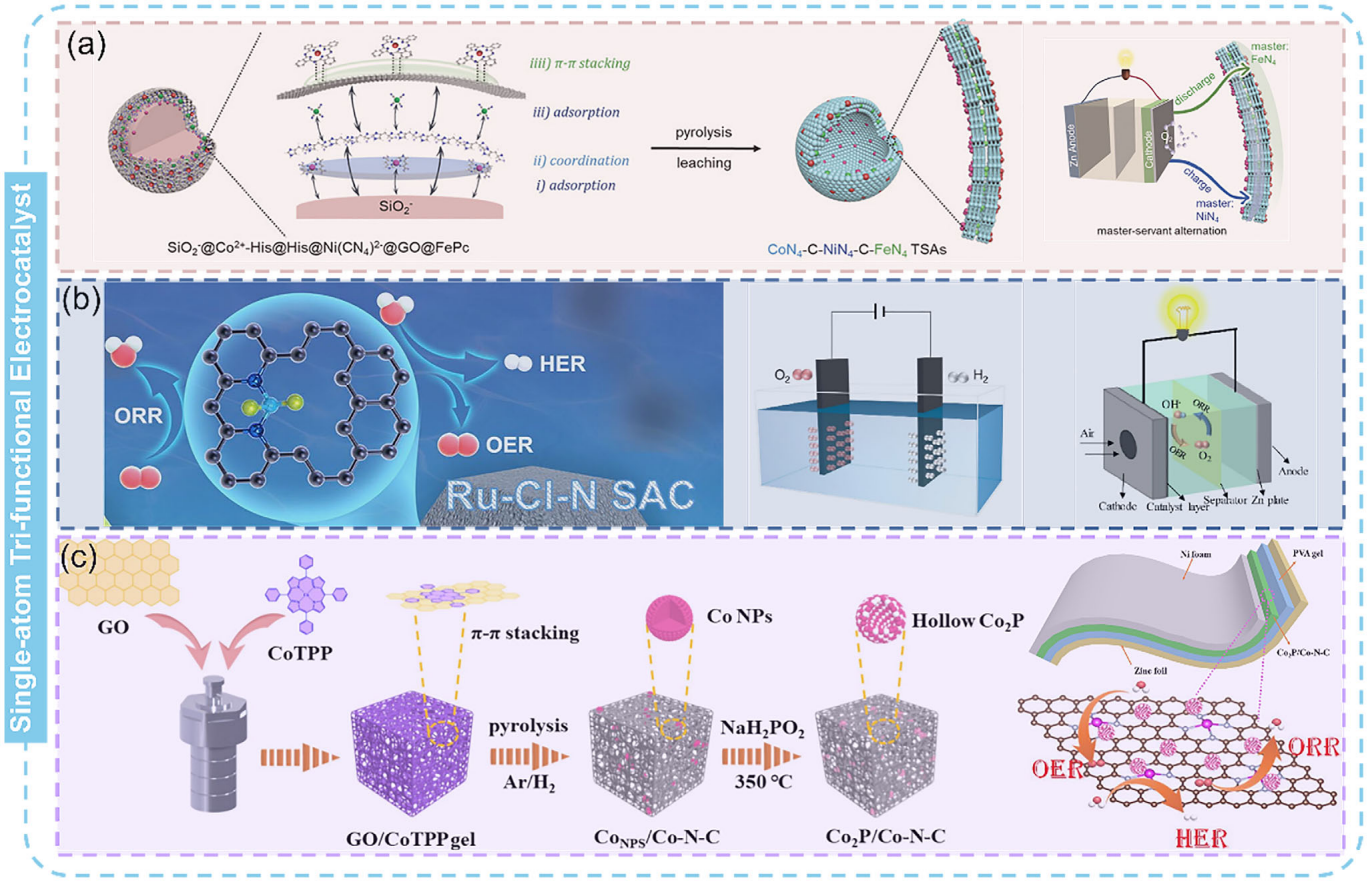
Single‐atom tri‐functional electrocatalyst. (a) Reproduced with permission [97]. Copyright 2022, Wiley. (b) Reproduced with permission [82]. Copyright 2022, Elsevier. (c) Reproduced with permission [98]. Copyright 2024, Elsevier.

SACs for tri‐functional electrocatalysis achieve high site density through atomic dispersion and electronic tuning via coordination engineering. However, the field still faces common challenges: (1) metal loadings are typically <1 wt.%, limiting volumetric activity; (2) the dynamic evolution of single‑atom sites under operating conditions is rarely tracked by operando techniques; (3) most reports focus on half‑cell metrics without device integration; (4) synthetic reproducibility and scalability remain unproven. Future work should combine in situ/operando characterization (XAS, Raman, TEM) with high‑throughput screening to elucidate dynamic behavior and develop robust synthesis methods that balance atom dispersion with practical loading.

### 3.6 Comprehensive Comparison and Overview of Structure–Activity Relationships

Rational design of efficient tri‐functional electrocatalysts is essential for integrated energy technologies such as rechargeable ZABs and WS. This review outlines the field's evolution from two angles: material systems and design logic. Material systems have progressed from precious metals to abundant, low‑cost non‑precious metals (Fe, Co, Ni), and further to advanced architectures, bimetallic/alloy systems, heterostructures, and single‑atom catalysts, driven by the need for high performance, stability, and low cost. Design logic has evolved from early performance validation and device demonstration to atomic/electronic‑scale mechanistic elucidation, and now to a synergy between mechanism‑guided rational design and application‑oriented systems engineering. Tables 6 and 7 compare representative state‑of‑the‑art tri‐functional electrocatalysts and their key metrics. Among them, Table 6 specifically compiles the DFT calculation details (models, descriptors, and intermediate free energies for HER/OER/ORR) from the literature, enabling a semi‑quantitative comparison based on the rate‑determining step (RDS) energy barriers. As can be seen from Table 6, a unified descriptor is still lacking. DFT models and descriptors are diverse, and the calculated free energies often refer to different intermediates or different adsorption sites. This heterogeneity makes direct numerical comparisons extremely difficult. To address this issue, we propose a relative RDS barrier analysis framework, which compares catalysts based on their respective rate‐determining step energies (Δ*G* HER; Δ*G* OER; Δ*G* ORR). Although not perfect, this approach provides a practical benchmark for evaluating activity trends. Looking forward, the establishment of standardized DFT benchmarking protocols for tri‐functional electrocatalysts will accelerate the rational design of non‐precious metal catalysts. Importantly, achieving practical breakthroughs requires collaborative optimization across three scales: atomic‑level electronic structure, nanoscale architecture, and macroscopic electrode integration.

**TABLE 6:** Summary of state‐of‐the‐art tri‐functional electrocatalysts: DFT data (2024‐2026).

Electrocatalyst	DFT model	Descriptor	Calculation type			Refs.
			Δ*G* HER (eV)	Δ*G* OER (eV)	Δ*G* ORR (eV)	
Co@NPCL	Co@nCNT(10%)	p‐band center	1.03 (Volmer step)	/	/	[99]
	nCNT(15%)		1.82	0.84 (*O→*OOH)	0.42 (O_2_→*OOH)	
	Co(111)@G		/	0.79 (*OH→*O)	0.06 (O_2_→*OOH)	
Co_2_P‐NCS‐*x*	Experimental only					[97]
Co_4_N@Fe/N‐C	Co_4_N@Fe/N‐C	d‐band center	1.04	/	/	[101]
Co_9_S_8_‐CoMo_2_S_4_/NSC	Experimental only					[86]
CoFe@NC/CoFe_2_O_4_/IF	Experimental only					[99]
Co/FeCo‐NCNT/NP	Experimental only					[100]
CoFe‐NiFe/NC	CoFe(110)/NiFe(111)@NC	d‐band center	/	1.73 (*O→*OOH)	0.48	[95]
CoMoO_4_/Co_3_O_4_@CC	CoMoO_4_/Co_3_O_4_(111)/CC	d‐band center	/	1.80 (*O→*OOH)	/	[89]
CoN NFs	Experimental only					[2]
Co_2_P/Co‐N‐C	Co_2_P/CoN_4_	d‐band center	/	/	0.48 (O*→H_2_O)	[98]
CoP@NC‐Ru	CoP(011)/N + Ru_4_	Bader charge	0.11	/	/	[104]
CO/R^0^/RO	Experimental only					[11]
Co‐S‐INF	Experimental only					[93]
CuFe_2_O_4_	Experimental only					[102]
Fe_3_C‐Fe/NC‐800	Fe_3_C(031)/Fe‐N_4_	/	/	/	0.351	[20]
LaCoO_3_/NiFe LDH	Experimental only					[61]
NC@CoNi	NC@Co_7_Ni_2_ (hcp/fcc)	/	/	2.53	/	[13]
NCMP	Ni‐Co‐Mn‐P	/	0.85	−0.20	/	[18]
NiFe_2_O_4_/IF	NiFe_2_O_4_(311)	/	0.77		/	[106]
NiMOF‐Ni_2_P/IF	NiMOF(110)/Ni_2_P(111)	d‐band center	/	1.96 (*O→*OOH)	/	[60]
NiO/IrO_2_‐NF	NiO(012)/IrO_2_(110)	/	0.099	/	/	[107]
RuSe_2_CoSe_2_/NC	RuSe_2_(210)/(200) + CoSe_2_(210)/(200)	/	0.06	/	/	[108]
V_O_‐CoWO_4_	CoWO_4_(010)	/	/	1.69 (*O→*OOH)	/	[90]
FeNi/FeNiO‐NCS	FeNi_3_(111)/Ni‐Fe_3_O_4_(311)/NC	d‐band center	−0.33	0.24 (*O→*OOH)	/	[93]
FeP/IF	Experimental only					[78]
Co_5.47_N/VN@NCFs	Co_5.47_N(111)/VN(200)	d‐band center	−0.03	/	/	[37]
Co_9_S_8_/CuCo_2_S_4_	Co_9_S_8_(440)/CuCo_2_S_4_(022)	Density of states	/	/	/	[94]
NiOOH/P‐MoS_2_/CC	Ni(OH)_2_/P‐MoS_2_	d‐band center	0.18	/	/	[109]
NCO/CO‐500	Experimental only					[13]
CoW‐N/C	Experimental only					[108]
RuCu/CoCu/C	Experimental only					[4]
FNCO‐600	Experimental only					[32]
TWCH‐Pt/MWCNTs	Pt/TiO*_x_*/MWCNTs	/	0.56	/	0.3 (*OH→OH^−^)	[29]
g‐C_3_N_4_‐PPy@CNR	Experimental only					[66]
Co‐Ni/NiF_2_@PCNFs	Co‐Ni/NiF_2_	d‐band center	/	/	1.07 (*O_2_→OOH)	[39]
FeCo LDH/TiN/NC	Experimental only					[42]
GF/Co@NCNTs	GF/Co@NCNT	/	/	1.66 (*O→*OOH)	1.66 (*O→*OOH)	[25]
NiFe‐Co/NC@NMO/NF	NiFe/Co/NC/NiMoO_4_	Density of states	/	1.51 (*O→*OOH)	/	[111]
NiCo_2_S_4_@GA/NF	NiCo_2_S_4_/Co_3_S_4_/NiS	d‐band center	/	0.24 (*OO →O_2_)	/	[112]
Mo‐NiFe_2_O_4_/IF	Experimental only					[88]
FeNi‐LDH/CA	Experimental only					[63]
CoNC_HS_/Cu_NWs_	Experimental only					[84]
GO*_x_*/N‐CNTs/GCE	Experimental only					[111]
Pt/Ni_3_(PO_4_)_2_/NF	Pt/Ni_3_(PO_4_)_2_	d‐band center	0.14	0.84	/	[24]
Zn‐FeCo@CNF‐900	FeCo/Fe(Zn)Co/FeCo(Zn)	d‐band center	/	0.15 (*O→*OOH)	0.16	[19]
Cu_0.81_Ni_0.19_@CoN/Mo_2_N	Cu_0.81_Ni_0.19_(111)/CoN(111)/Mo_2_N(111)	Bader charge	0.65	/	/	[36]
FeCoPP/G	FeCoPP	DOS	0.11	/	/	[26]
NFS‐XC/72	N,F,S‐doped carbon	DOS	0.41	2.70	/	[28]

**TABLE 7:** Summary of state‐of‐the‐art tri‐functional electrocatalysts: experimental data (2024–2026).

Electrocatalyst	Activity and performance	Δ*E* (V)^a^	Power density (mW/cm^2^)	Specific capacity (mAh/g)	H_2_ evolution rate (µL/s)	Device validation	Refs.
Pt/C (20 wt.%)	*η*_10_HER = 25 mV^b^, *E*_1/2_ORR = 0.88V^c^	/	/	/	/	Commercial	[72]
IrO_2_	*η*_10_OER = 360 mV^c^	/	/	/	/	Commercial	[73]
Co@NPCL	*η*_10_HER = 87 mV^b^, *η*_10_OER = 276 mV^b^, *E*_1/2_ORR = 0.86 V^c^, *η*_10_WS = 1.58 V^c^	0.65	135^d^	833	/	Standalone device	[99]
Co_2_P‐NCS‐x	*η*_10_HER = 193 mV^b^, *η*_10_OER = 292 mV^b^, *E*_1/2_ORR = 0.89 V^c^, *η*_10_WS = 1.69 V^b^	0.63	219^d^	/	3.4	Full device	[100]
Co_4_N@Fe/N‐C	*η*_10_HER = 270 mV^b^, *η*_10_OER = 310 mV^b^, *E*_1/2_ORR = 0.76 V^c^, *η*_10_WS = 1.76 V^b^	0.78	104.5^d^	789.9	/	Full device	[101]
Co_9_S_8_‐CoMo_2_S_4_/NSC	*η*_10_HER = 89 mV^b^, *η*_10_OER = 280 mV^b^, *E*_1/2_ORR = 0.86 V^c^, *η*_10_WS = 1.55 V^b^	0.65	112.82^d^	804	/	Full device	[42]
CoFe@NC/CoFe_2_O_4_/IF	*η*_10_HER = 66 mV^b^, *η*_10_OER = 185 mV^b^, *E*_1/2_ORR = 0.85 V^c^, *η*_10_WS = 1.50 V^b^	0.57	134.5^d^	/	/	Full device	[40]
Co/FeCo‐NCNT/NP	*η*_10_HER = 228 mV^b^, *η*_10_OER = 339 mV^b^, *E*_1/2_ORR = 0.91 V^c^, *η*_10_WS = 1.67 V^b^	0.66	220.2^d^	814.2	/	Standalone device	[102]
CoFe‐NiFe/NC	*η*_10_HER = 66 mV^b^, *η*_10_OER = 185 mV^b^, *E*_1/2_ORR = 0.81 V^c^, *η*_10_WS = 1.51 V^b^	0.61	155^d^	809@10	23.5	Full device	[95]
CoMoO_4_/Co_3_O_4_@CC	*η*_10_OER = 250 mV^b^, *η*_10_WS = 1.51 V^b^	/	120.3^d^	654	/	Standalone device	[89]
CoN NFs	*η*_10_HER = 188 mV^b^, *η*_10_OER = 282 mV^b^, *E*_1/2_ORR = 0.82 V^c^, *η*_10_WS = 1.61 V^b^	0.69	107.3^d^	/	/	Standalone device	[103]
Co_2_P/Co‐N‐C	*η*_10_HER = 190 mV^b^, *η*_10_OER = 330 mV^b^, *E*_1/2_ORR = 0.89 V^c^	0.67	91^f^	700	/	Full device	[98]
CoP@NC‐Ru	*η*_10_HER = 35 mV^b^, *η*_10_OER = 270 mV^b^, *E*_1/2_ORR = 0.89 V^c^, *η*_10_WS = 1.47 V^b^	0.61	65^d^	780	/	Standalone device	[104]
CO/R^0^/RO	*η*_10_HER = 58 mV^b^, *η*_10_OER = 166 mV^b^, *E*_1/2_ORR = 0.94 V^c^, *η*_10_WS = 1.49 V^b^	0.46	376.4^d^	/	6.66	Full device	[12]
Co‐S‐INF	*η*_100_HER = 289 mV^b^, *η*_100_OER = 248 mV^b^, *E*_1/2_ORR = 0.68 V^c^	/	332.3^d^	798.2	/	Standalone device	[96]
CuFe_2_O_4_	*η*_10_HER = 342 mV^b^, *η*_10_OER = 684 mV^b^	/	/	/	/	Standalone device	[105]
Fe_3_C‐Fe/NC‐800	*η*_100_HER = 124 mV^b^, *η*_10_OER = 230 mV^b^, *E*_1/2_ORR = 0.86 V^c^, *η*_10_WS = 1.70 V^b^	0.60	/	813	/	Full device	[20]
LaCoO_3_/NiFe LDH	*η*_10_OER = 260 mV^b^	/	/	/	/	Standalone device	[65]
NC@CoNi	*η*_10_HER = 275 mV^b^, *η*_10_OER = 330 mV^b^, *E*_1/2_ORR = 0.87 V^c^, *η*_10_WS = 1.76 V^b^	0.69	95^d^	781@20	/	Standalone device	[13]
NCMP	*η*_10_HER = 100 mV^b^, *η*_50_OER = 218 mV^b^, *E*_1/2_ORR = 0.76 V^c^	/	148^g^	740.3	/	Standalone device	[18]
NiFe_2_O_4_/IF	*η*_100_HER = 115 mV^b^, *η*_100_OER = 331 mV^b^, *η*_10_WS = 1.52 V^b^	/	138.1^h^	/	/	Full device	[106]
NiMOF‐Ni_2_P/IF	*η*_100_OER = 240 mV^b^, *η*_100_WS = 1.53 V^b^	/	/	/	/	Standalone device	[60]
NiO/IrO_2_‐NF	*η*_10_HER = 42 mV^b^, *η*_10_OER = 240 mV^b^, *E*_1/2_ORR = 0.80 V^c^, *η*_10_WS = 1.51 V^b^	0.67	134.8^d^	726	/	Full device	[107]
RuSe_2_CoSe_2_/NC	*η*_10_HER = 31 mV^b^, *η*_10_OER = 248 mV^b^, *E*_1/2_ORR = 0.83 V^c^, *η*_10_WS = 1.57 V^b^	0.65	156.6^d^	/	/	Standalone device	[108]
V_O_‐CoWO_4_	*η*_10_OER = 260 mV^b^, *η*_10_WS = 1.50V	/	152.2^i^	573.2	/	Standalone device	[90]
FeNi/FeNiO‐NCS	*η*_10_HER = 210 mV^b^, *η*_10_OER = 300 mV^b^, *E*_1/2_ORR = 0.86 V^c^, *η*_10_WS = 1.59 V^b^	0.67	156^d^	855	/	Full device	[93]
FeP/IF	*η*_100_HER = 242 mV^b^, *η*_100_OER = 397 mV^b^, *η*_10_WS = 1.67 V^b^	/	/	250	/	Full device	[84]
Co_5.47_N/VN@NCFs	*η*_10_HER = 89 mV^b^, *η*_10_OER = 290 mV^b^, *E*_1/2_ORR = 0.79 V^c^, *η*_10_WS = 1.53 V^b^	0.73	207^d^	789	/	Full device	[37]
Co_9_S_8_/CuCo_2_S_4_	*η*_10_HER = 191 mV^b^, *η*_10_OER = 264 mV^b^, *E*_1/2_ORR = 0.79 V^c^, *η*_10_WS = 1.66 V^b^	0.70	89.24^d^	706.6	/	Full device	[94]
NiOOH/P‐MoS_2_/CC	*η*_10_HER = 90 mV^b^ *, η*_10_OER = 220 mV^b^, *E*_1/2_ORR = 0.79 V^c^, *η*_10_WS = 1.42 V^b^	0.70	125^d^	760	/	Standalone device	[109]
NCO/CO‐500	*η*_10_HER = 163 mV^b^, *η*_10_OER = 274 mV^b^, *E*_1/2_ORR = 0.76 V^c^ *, η*_10_WS = 1.72 V^b^	0.74	/	/	/	Standalone device	[14]
CoW‐N/C	*η*_10_HER = 242 mV^b^, *η*_10_OER = 1.60 V^b^, *E*_1/2_ORR = 0.81 V^c^, *η*_10_WS = 1.79 V^b^	0.79	111.3^d^	940	/	Standalone device	[64]
RuCu/CoCu/C	*η*_10_HER = 11 mV^b^, *η*_10_OER = 480 mV^b^, *E*_1/2_ORR = 0.74 V^c^, *η*_10_WS = 1.51 V^b^	0.79	234^d^	/	/	Standalone device	[110]
FNCO‐600	*η*_10_HER = 211 mV ^b^, *η*_10_OER = 230 mV^b^, *E*_1/2_ORR = 0.76 V^c^, *η*_10_WS = 1.72 V^b^	0.70	21.8^d^	973	/	Standalone device	[33]
TWCH‐Pt/MWCNTs	*η*_10_HER = 60 mV^b^, *η*_10_OER = 313 mV^c^, *E*_1/2_ORR = 0.89 V^b^	0.65	227^d^	/	/	Full device	[29]
g‐C_3_N_4_‐PPy@CNR	*η*_10_OER = 320 mV^b^, *E*_1/2_ORR = 0.92 V^c^	0.65	125.23^d^	541.2	/	Full device	[71]
Co‐Ni/NiF_2_@PCNFs	*η*_10_OER = 257 mV^b^, *E*_1/2_ORR = 0.84 V^c^	0.63	271^d^	778.9	/	Standalone device	[39]
FeCo LDH/TiN/NC	*η*_10_HER = 238 mV^b^, *η*_10_OER = 303 mV^b^, *E*_1/2_ORR = 0.90 V^c^, *η*_10_WS = 1.57 V^b^	0.65	92^d^	830.3	/	Standalone device	[44]
GF/Co@NCNTs	*η*_10_HER = 195 mV^b^, *η*_10_OER = 260 mV^c^, *E*_1/2_ORR = 0.84 V^b^, *η*_10_WS = 1.59 V^b^	0.63	78.33^d^	718.05	16.33	Full device	[24]
NiFe‐Co/NC@NMO/NF	*η*_100_HER = 214 mV^b^ *, η*_100_OER = 298 mV^b^, *E*_1/2_ORR = 0.86 V^c^, *η*_10_WS = 1.54 V^b^	/	104.4^d^	743	/	Standalone device	[111]
NiCo_2_S_4_@GA/NF	*η*_50_OER = 194 mV^b^	/	20^d^	/	/	Standalone device	[112]
Mo‐NiFe_2_O_4_/IF	*η*_100_HER = 311.7 mV^b^, *η*_100_OER = 275.4 mV^b^, *η*_10_WS = 1.69 V^b^	/	106^j^	/	/	Full device	[91]
FeNi‐LDH/CA	*η*_10_OER = 250 mV^b^, *η*_10_WS = 1.48 V^b^	/	105^d^	/	/	Standalone device	[67]
CoNC_HS_/Cu_NWs_	*η*_20_HER = 163 mV^b^, *η*_20_OER = 289 mV^b^, *η*_10_WS = 1.63 V^b^	/	46	/	/	Standalone device	[38]
GOx/N‐CNTs/GCE	*η*_100_OER = 240 mV^b^, *η*_10_WS = 1.60 V^b^	/	6.79	/	/	Standalone device	[113]
Pt/Ni_3_(PO_4_)_2_/NF	*η*_100_HER = 38 mV^b^, *E*_1/2_ORR = 0.88 V^c^, *η*_10_WS = 1.58 V^b^	/	153	780.35	/	Full device	[24]
Zn‐FeCo@CNF‐900	*η*_10_HER = 169 mV^b^, *η*_10_OER = 268 mV^b^, *E*_1/2_ORR = 0.84 V^c^, *η*_10_WS = 1.68 V^b^	0.67	195	735.6	/	Full device	[19]
Cu_0.81_Ni_0.19_@CoN/Mo_2_N	*η*_10_HER = 43 mV^b^, *η*_10_OER = 297 mV^b^, *E*_1/2_ORR = 0.88 V^c^, *η*_10_WS = 1.56 V^b^	0.65	81.72	811.6	/	Full device	[36]
FeCoPP/G	*η*_10_HER = 79mV ^b^, *η*_10_OER = 255 mV^c^, *E*_1/2_ORR = 0.87 V^b^, *η*_10_WS = 1.61 V^b^	0.62	237	753	/	Full device	[26]
NFS‐XC/72	*η*_10_HER = 296 mV^c^, *η*_10_OER = 370 mV^b^, *E*_1/2_ORR = 0.83 V^c^, *η*_10_WS = 1.70 V^b^	0.77	201.7	804.3	/	Standalone device	[28]

Measurement conditions:

^a^ Δ*E* = *E* **_{OER, j = 10}_ −** *E* **_{ORR, 1/2}_** [74].

^b^ 1 m KOH.

^c^ 0.1 m KOH.

^d^ 6 m KOH+0.2m Zn(OAc)_2_.

^f^ PVA‐KOH.

^g^ 6 m KOH + ZnO.

^h^ 6 m KOH + 0.02 m Zn(OAc)_2_.

^i^ 6.0 m KOH + 0.2 m Zn(Ac)_2_ + 0.5 m K_2_WO_4_.

^j^ 6 m KOH+0.02m Zn(OAc)_2_.

Full device: the catalyst has been tested in an integrated ZAB‐driven water splitting system. Standalone device: the catalyst has been tested in a ZAB or electrolyzer separately, but the coupled system data are missing.

### 3.7 Insights From Operando Spectroscopy on Catalyst Stability and Degradation

The preceding sections have systematically reviewed the design strategies, material systems, and structure–activity relationships of tri‐functional electrocatalysts. However, evaluating catalyst performance solely based on half‐cell metrics often neglects the stability challenges inherent in practical device operations. Given that the discussions on sulfide/phosphide reconstruction, metal dissolution, and the scarcity of operando evidence remain fragmented in previous sections, this section aims to systematically consolidate these observations, summarize the deactivation patterns, and emphasize how operando spectroscopy provides robust experimental evidence for elucidating these degradation mechanisms.

Metal oxides or selenides undergo surface reconstruction under OER, requiring a strict distinction between beneficial surface evolution and irreversible bulk degradation. Zhu et al. [89] (Figure 11a,b) illustrated an ideal case, where selective Mo etching from CoMoO_4_ generates an amorphous CoOOH active phase in situ, while the underlying crystalline Co_3_O_4_ skeleton remains dynamically stable, forming an efficient amorphous‐crystalline heterojunction. Li et al. [108] (Figure 11c) confirmed this via in situ Raman, showing the disappearance of Co─Se/Ru─Se bonds and the simultaneous rise of CoOOH peaks with increasing potential. Nevertheless, once reconstruction propagates into the bulk, the rigid conductive scaffold collapses, leading to structural pulverization, loss of electrical pathways, and rapid catalyst failure.

**FIGURE 11:**
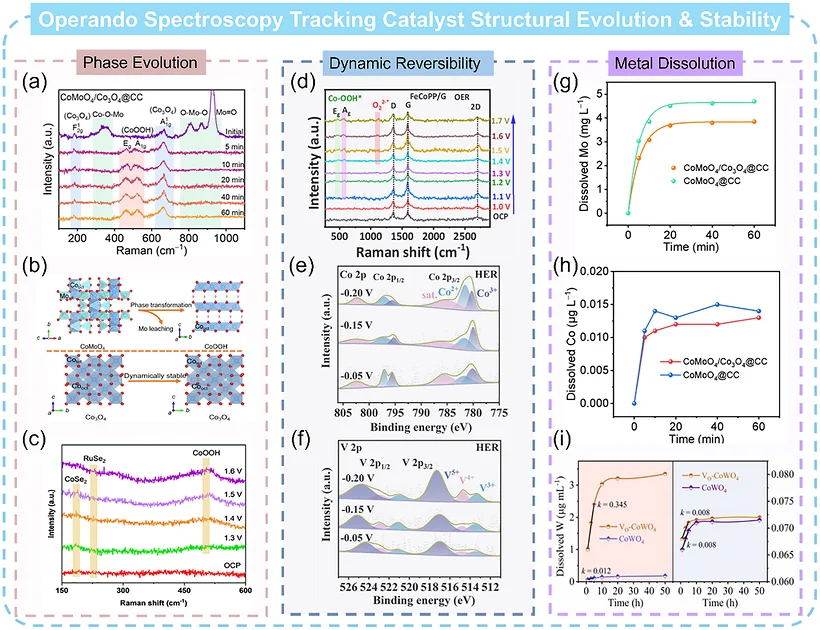
Operando spectroscopy on catalyst stability and degradation. (a) In situ Raman spectra of CoMoO_4_/Co_3_O_4_@CC. (b) A schematic diagram of the selective reconstruction of CoMoO_4_/Co_3_O_4_@CC during OER. Reproduced with permission [89]. Copyright 2024, Elsevier. (c) In situ Raman spectra during the OER process. Reproduced with permission [108]. Copyright 2024, ACS. (d) In situ Raman spectra of FeCoPP/G for OER. Reproduced with permission [26]. Copyright 2026, Elsevier. Quasi‐operando XPS spectra of Co_5.47_N/VN@NCFs at different potentials, where (e,f) represent the variations in the Co 2p and V 2p spectra during the HER. Reproduced with permission [37]. Copyright 2025, Elsevier. The leaching concentrations of (g) Mo and (h) Co ions of CoMoO_4_/Co_3_O_4_@CC and CoMoO_4_@CC in the electrolyte during the CA under 1 m KOH. Reproduced with permission [89]. Copyright 2024, Elsevier. (i) The concentrations of leached W and Co detected by ICP and the linear fitting results according to the Noyes–Whitney equation. Reproduced with permission [90]. Copyright 2024, Elsevier.

Operando spectroscopy provides direct experimental evidence for dynamic reversibility. Kumar et al. [26] (Figure 11d) utilized in situ Raman spectroscopy to reveal the dynamic generation and consumption of O_2_* and OOH* intermediates during the ORR; Zhang et al. [37] (Figure 11e,f) further demonstrated through quasi‐operando XPS that the electronic compensation effect of VN drives the fully reversible switching of Co and V valence states (e.g., Co^2+^/Co^3+^, V^4+^/V^5+^) under alternating potentials. The transient response of these reaction intermediates, coupled with the electronic reversibility of the active‐site valence states, constitutes the core criterion for catalysts to resist irreversible deep oxidation and maintain long‐term anti‐deactivation capability under alternating operating conditions.

Quantitative inductively coupled plasma (ICP) analysis clearly delineates the pronounced selectivity of metal dissolution. Zhu et al. [89] (Figure 11g,h) confirmed that the inactive Mo species in CoMoO_4_/Co_3_O_4_@CC undergo rapid leaching within 20 min (∼4.6 mg/L), whereas the loss of active Co sites remains consistently negligible (<0.02 µg/L). Zhu et al. [90] (Figure 11i) further corroborated this trend in the V_O_‐CoWO_4_ system, where the initial W leaching rate was approximately 30 times higher than that of CoWO_4_, yet the dissolution of Co remained negligible. Notably, this selective and rapid dissolution of inactive framework elements not only compromises the mechanical integrity and electronic transport pathways of the conductive network, but also directly triggers physical failure and mass transport blockage at the gas–liquid–solid triple‐phase boundary on a macroscopic scale, constituting the fundamental driving force for rapid catalyst deactivation under frequent operational cycling.

Collectively, operando spectroscopy not only reveals the structural evolution and degradation pathways of catalysts under operational conditions but also uncovers the core mechanisms for circumventing irreversible deactivation. Through distinguishing beneficial reconstruction from irreversible degradation, monitoring valence‐state reversibility, and quantitatively tracking metal dissolution, the fundamental criteria for long‐term stability are clarified. Nevertheless, the stability of microscopic active sites does not guarantee the stability of macroscopic devices.

### 3.8 Multidimensional Evaluation and Performance‐Practicality Trade‐Offs of Tri‐Functional Electrocatalysts

Beyond intrinsic activity, practical device applications necessitate a comprehensive evaluation of cost, activity, stability, and scalability. Based on a literature survey, Table 8 provides a qualitative and semi‐quantitative summary of five representative catalyst systems across these four dimensions. Among them, metal‐free catalysts offer the lowest cost, yet their activity and stability are constrained by the inherent susceptibility of the carbon skeleton to corrosion at high potentials; noble metal‐based catalysts exhibit superior activity, but their extremely low abundance and high cost severely limit long‐term applications; transition metal‐based catalysts balance cost and activity, yet their dynamic reconstruction and metal dissolution readily trigger triple‐phase boundary degradation, leading to a serious stability crisis. Heterojunction catalysts achieve excellent synergistic activity through interfacial charge modulation; however, their complex preparation processes and interfacial matching pose severe challenges to scalability. Single‐atom catalysts theoretically possess the highest atom utilization efficiency, but their actual ultra‐low metal loading makes it difficult to translate into volumetric activity, and high‐temperature agglomeration issues impede large‐scale synthesis.

**TABLE 8:** Comprehensive performance comparison of tri‐functional electrocatalysts (2024–2026).

Catalyst type	Electrocatalyst	Cost	Activity (Δ*E*)	Stability (*h*)	Scalability	Refs.
Metal‐free	NFS‐XC/72	Very low	0.77	1360 (ZAB)	Excellent	[28]
	g‐C_3_N_4_‐PPy@CNR	Very low	0.65	90 (ZAB)	Excellent	[71]
Noble metal‐based	TWCH‐Pt/MWCNTs	High (Pt)	0.65	365 (ZAB)	Excellent	[29]
	Pt/Ni_3_(PO_4_)_2_/NF	High (Pt)	/	1500 (ZAB)	Good	[24]
	CO/R^0^/RO	Medium (Ru)	0.46	2000 (ZAB)	Good	[12]
	RuCu/CoCu/C	Medium (Ru)	0.79	1000 (ZAB)	Good	[110]
	RuSe_2_CoSe_2_/NC	Medium (Ru)	0.65	140 (ZAB)	Good	[108]
	CoP@NC‐Ru	Low‐medium (Ru)	0.61	50 (ZAB)/25 (WS)	Good	[104]
	NiO/IrO_2_‐NF	High (Ir)	0.67	100 (ZAB)/600 (WS)	Good	[107]
Transition‐metal	Co@NPCL	Low (Co)	0.65	250 (ZAB)	Good	[99]
	Co_2_P‐NCS‐*x*	Low (Co)	0.63	60 (ZAB)	Good	[100]
	Co_4_N@Fe/N‐C	Low (Co, Ni)	0.78	500 cycles (ZAB)	Good	[101]
	Co_9_S_8_‐CoMo_2_S_4_/NSC	Low (Co, Mo)	0.65	97 (ZAB)/30 (WS)	Excellent	[42]
	CoFe@NC/CoFe_2_O_4_/IF	Low (Co, Fe)	0.57	210 (ZAB)	Good	[40]
	Co/FeCo‐NCNT/NP	Low (Co, Fe)	0.66	100 (ZAB)	Good	[102]
	CoFe‐NiFe/NC	Low (Co, Fe, Ni)	0.61	400 (ZAB)/27 (WS)	Good	[95]
	CoMoO_4_/Co_3_O_4_@CC	Low (Co, Mo)	/	1200 (ZAB)/500 (WS)	Good	[89]
	CoN NFs	Low (Co)	0.69	30 (WS)	Good	[103]
	CuFe_2_O_4_	Low (Cu, Fe)	/	7 (OER/HER)	Good	[105]
	Fe_3_C‐Fe/NC‐800	Low (Fe)	0.60	120 (ZAB)/90 (WS)	Good	[20]
	NiFe_2_O_4_/IF	Low (Ni, Fe)	/	1600 (ZAB)/180 (WS)	Good	[106]
	V_O_‐CoWO_4_	Low (Co,W)	/	1389 (ZAB)	Good	[90]
	FeP/IF	Low (Fe)	/	250 (ZAB)	Good	[84]
	Co_5.47_N/VN@NCFs	Low (Co, V)	0.73	800 (ZAB)	Good	[37]
	NiOOH/P‐MoS_2_/CC	Low (Ni, Mo)	0.7	300 (ZAB)	Good	[109]
	NCO/CO‐500	Low (Ni, Co)	0.74	120 (ZAB)	Good	[14]
	CoW‐N/C	Low (Co, W)	0.79	50 (ZAB)	Good	[64]
	FNCO‐600	Low (Fe, Ni, Co)	0.70	70 (ZAB)	Good	[33]
	NiCo_2_S_4_@GA/NF	Low (Ni, Co)	/	1500 (ZAB)	Good	[112]
	Mo‐NiFe_2_O_4_/IF	Low (Ni, Fe, Mo)	/	700 (ZAB)	Good	[91]
	FeNi‐LDH/CA	Low (Ni, Fe)	/	200 (ZAB)	Good	[67]
	CoNC_HS_/Cu_NWs_	Low (Co, Cu)	/	200 (ZAB)	Good	[38]
	Zn‐FeCo@CNF‐900	Low (Zn, Fe, Co)	0.67	600 (ZAB)	Good	[19]
	FeCoPP/G	Low (Fe, Co)	0.62	160 (ZAB)	Good	[26]
Heterostructure	Co‐S‐INF	Low (Co, Fe, Ni)	/	12(ZAB)/27(WS)	Good	[96]
	LaCoO_3_/NiFe LDH	Medium (La, Co, Ni, Fe)	/	30(ZAB)	Good	[61]
	NC@CoNi	Low (Co, Ni)	0.69	200(ZAB)	Good	[13]
	NCMP	Low (Ni, Co, Mn)	/	120 (WS)	Good	[18]
	NiMOF‐Ni_2_P/IF	Low (Ni)	/	200 (ZAB)/200 (WS)	Good	[60]
	FeNi/FeNiO‐NCS	Low (Fe, Ni)	0.67	1350 (ZAB)	Good	[93]
	Co_9_S_8_/CuCo_2_S_4_	Low (Co, Cu)	0.70	450 (ZAB)	Good	[94]
	Co‐Ni/NiF_2_@PCNFs	Low (Co, Ni)	/	600 (ZAB)	Good	[39]
	FeCo LDH/TiN/NC	Low (Fe, Co, Ti)	0.65	200 (ZAB)	Good	[44]
	GF/Co@NCNTs	Low (Co)	0.63	375 (ZAB)	Good	[24]
	NiFe‐Co/NC@NMO/NF	Low (Ni, Fe, Co)	/	150 (ZAB)	Good	[111]
	GOx/N‐CNTs/GCE	Low	/	200 (ZAB)	Good	[113]
	Cu_0.81_Ni_0.19_@CoN/Mo_2_N	Low (Cu, Ni, Co, Mo)	0.65	150 (ZAB)	Good	[36]
Single‐atom	Co_2_P/Co‐N‐C	Low	0.67	80 (ORR)	Good	[98]

*Note*: Δ*E* = *E* **_{OER, j = 10}_ −** *E* **_{ORR, 1/2}_** [74]. The qualitative ratings in the table are based on a semi‐quantitative cross‐literature assessment and are intended to reflect general trends across catalyst systems rather than absolute numerical rankings, given the inherent variations in testing conditions across reports. Notably, the assessment of “Scalability” is not an isolated metric but a qualitative conclusion that comprehensively considers multiple factors, including raw material cost, synthesis complexity, energy consumption, scale‐up safety, and performance reproducibility.

This cross‐system comparison clearly reveals that no single catalyst system can currently excel simultaneously across all four dimensions; instead, significant performance‐practicality trade‐offs are prevalent among different systems. Therefore, future catalyst design should not be confined to the extreme pursuit of single overpotential metrics but should pivot toward a Pareto‐based multidimensional synergy that accounts for activity, stability, cost, and scalability, thereby truly bridging the gap between intrinsic material performance and macroscopic device applications.

## 4 Integrated Applications: From Single Devices to Self‐Powered Energy Systems

The ultimate value of a tri‐functional electrocatalyst lies not in half‑cell overpotentials or Tafel slopes, but in stable, efficient operation within real devices. However, no linear correlation exists between RDE performance and device‑level behavior [46, 114]. A catalyst that excels in a three‑electrode cell often underperforms in a ZAB, water electrolyzer, or self‑powered system combining both. This disconnect arises because different devices impose conflicting requirements: gas‑‐liquid‑‐solid triple‑phase interfaces in ZABs versus bubble‑prone, high‑current surfaces in electrolyzers; alternating ORR/OER in air electrodes versus continuous HER/OER in WS. Moreover, when a ZAB drives water electrolysis, system performance is governed by voltage/power matching, capacity decay, and dynamic load response, not single‑reaction metrics.

This section first evaluates tri‐functional catalysts in ZABs and WS devices, then analyzes ZAB‑driven self‑powered systems, and finally explores solar‑assisted, flexible, and reconfigurable configurations [115]. A crucial feature of our work is the feedback loop linking device performance to catalyst properties, which is overlooked in most reviews.

### 4.1 Applications of Tri‐Functional Electrocatalysts in Rechargeable ZAB and WS Devices

In rechargeable ZAB, tri‐functional electrocatalysts primarily serve as air electrode catalysts, catalyzing ORR during discharge and OER during charging. HER activity is not directly used here but becomes critical when the same catalyst is later employed in WS [116]. Therefore, when assessing tri‐functional catalysts for ZABs, the focus must be on ORR/OER bifunctionality, three‑phase interface stability, and cyclability during charge‑discharge, not on half‑cell potentials. During discharge, inappropriate adsorption of O_2_, OOH*, O*, or OH* can trigger a two‐electron pathway, generating peroxides that degrade efficiency and accelerate electrode failure [36, 117]. During charge, high anodic potential may corrode carbon supports, restructure metal active phases, leach metal ions, and delaminate the catalytic layer. Thus, ZABs demand more robust catalysts than those for single ORR or OER.

Performance metrics for ZABs should include open‑circuit voltage, peak power density, specific capacity, energy density, charge‐‑discharge voltage gap, round‑trip efficiency, and cycle life [110, 118]. Post‑test characterization (metal valence, surface oxide, carbon corrosion, pore blockage) is equally important, as these changes affect subsequent water‑splitting performance.

Compared to ZAB, WS device electrolysis cells impose a different set of requirements on tri‐functional catalysts. The catalyst must catalyze HER at the cathode and OER at the anode. Cathodic HER typically requires the catalyst to possess a moderate H* adsorption free energy and high‐efficiency water molecule dissociation capability, while OER at the anode requires the catalyst to effectively regulate oxygen‐containing intermediates such as OH*, O*, and OOH*, and to remain stable at high oxidation potentials [44, 111]. Here, the catalyst drives HER (cathode) and OER (anode). Transition metal sulfides, phosphides, nitrides, and carbides often undergo surface reconstruction under OER conditions, forming active phases such as metal hydroxides [29]. This may enhance OER but can destroy the original structure, reduce conductivity, or compromise HER/ORR activity. Therefore, evaluation must include two‑electrode polarization, long‑term stability, faradaic efficiency, and post‑operation phase analysis.

It is worth noting that the optimal operating conditions for the same catalyst often differ between ZAB and WS systems [119, 120]. For example, the air electrode in a ZAB requires the formation of a stable gas–liquid–solid three‐phase interface to balance oxygen diffusion, electrolyte wetting, and electron transport, whereas WS electrodes place greater emphasis on sufficient electrolyte contact, rapid bubble desorption, and structural stability under high current densities. There may be significant differences in the optimal conditions for both systems regarding catalyst loading, electrode thickness, pore structure, hydrophilicity/hydrophobicity, binder ratio, and substrate selection [121, 122]. Furthermore, the surface species formed on the catalyst during the charging and discharging of ZAB may not necessarily represent the optimal active phase for use in water‐splitting electrolytic cells [24, 123]. This issue of adapting a single material to multiple operating conditions has not been fully addressed in many reports, yet it constitutes the primary challenge for the integrated application of tri‐functional catalysts.

### 4.2 Self‐Powered Water‐Splitting System Driven by a Zinc–Air Battery

Coupling a ZAB with a water‐splitting electrolyzer to construct a self‐powered water‐splitting system is one of the most representative integrated applications of tri‐functional electrocatalysts [114, 124]. Conceptually, this strategy achieves the coupling of energy storage and hydrogen production, offering new insights for distributed hydrogen production and portable energy systems. However, from an engineering perspective, using a ZAB to drive water electrolysis is not simply a matter of connecting devices in series; rather, it involves a complex balancing act between voltage, power, capacity, and stability.

Voltage matching is one of the core challenges facing self‐powered WS systems [125, 126]. The actual operating voltage of a single ZAB is typically around 1.2–1.4 V, whereas alkaline WS electrolyzers generally require a drive voltage of approximately 1.6–1.8 V or even higher to achieve a substantial current density. This voltage difference makes it difficult for a single ZAB to sustainably drive efficient WS [127, 128]. Consequently, existing research typically employs two or more ZABs connected in series to achieve sufficient output voltage. However, while series battery packs can increase the total voltage, they also introduce issues such as additional internal resistance, performance inconsistencies between cells, and energy losses. When the battery pack's output voltage significantly exceeds the voltage required by the electrolyzer, excess energy may be dissipated as heat or lost due to polarization, thereby reducing the system's overall energy efficiency.

In addition, power matching and the dynamic operating point also determine the system's actual performance. The output voltage of a ZAB is not a constant value; rather, it varies continuously with discharge time, discharge current, the state of the zinc anode, electrolyte concentration, and air electrode polarization [129]. At the same time, the operating current of the electrolyzer is determined by its polarization curve and is highly sensitive to changes in input voltage. Therefore, the actual operating point of a self‐powered system should be determined by the intersection of the ZAB discharge polarization curve and the electrolyzer polarization curve, rather than simply by the open‐circuit voltage or the theoretical voltage for water electrolysis [130]. The practical operating point is determined by the intersection of the ZAB discharge curve and the electrolyzer polarization curve. However, the testing conditions for these two polarization curves vary considerably across the current literature, making reliable cross‐literature quantitative intersection reconstruction difficult to achieve. We therefore recommend that complete polarization curves and their operating‐point intersection analysis be established as key parameters for standardized reporting in future studies. As the voltage gradually decreases during the discharge of the ZAB, this intersection point continuously shifts, leading to dynamic changes in the current and hydrogen production rate. When the battery output voltage falls below the cell's startup voltage or maintenance voltage, the gas production rate will decrease significantly or even cease.

Currently, many reports on ZAB driving WS remain at the feasibility demonstration stage, typically using photographs of lit LEDs, bubble formation, or short‐term gas production as evidence of successful operation of the integrated system [131]. However, such qualitative demonstrations fail to reflect the system's true energy conversion capability and long‐term stability. For practical applications, more critical parameters should include self‐powered startup voltage, steady‐state operating current, continuous runtime, hydrogen yield, hydrogen production Faradaic efficiency, ZAB capacity utilization, overall system energy efficiency, and the curve of hydrogen production rate versus discharge time [132, 133]. Dynamic hydrogen production behavior over a complete discharge cycle, the system's recovery capability following battery replacement or recharging, and the mechanisms of interface degradation after multiple cycling operations remain underreported in current research.

Based on the device connection method, existing ZAB‐powered WS systems can be broadly classified into two categories. One category is the direct‐drive mode, in which one or more ZAB directly power the electrolyzer [12]. This configuration is simple and cost‐effective, making it suitable for proof‐of‐concept and miniaturized systems. The other category is the series battery pack drive mode, in which multiple ZABs are connected in series to increase the output voltage and meet the startup and operational requirements of the electrolyzer, as shown in (Figure 12a,b). This approach enhances drive capacity but also introduces issues such as voltage redundancy, increased internal resistance, and stricter battery consistency requirements. In the future, relying solely on increasing the number of batteries in series will not be an ideal solution for improving system efficiency; rather, it will require coordinated optimization across three levels: the catalyst, the electrodes, and the system.

**FIGURE 12:**
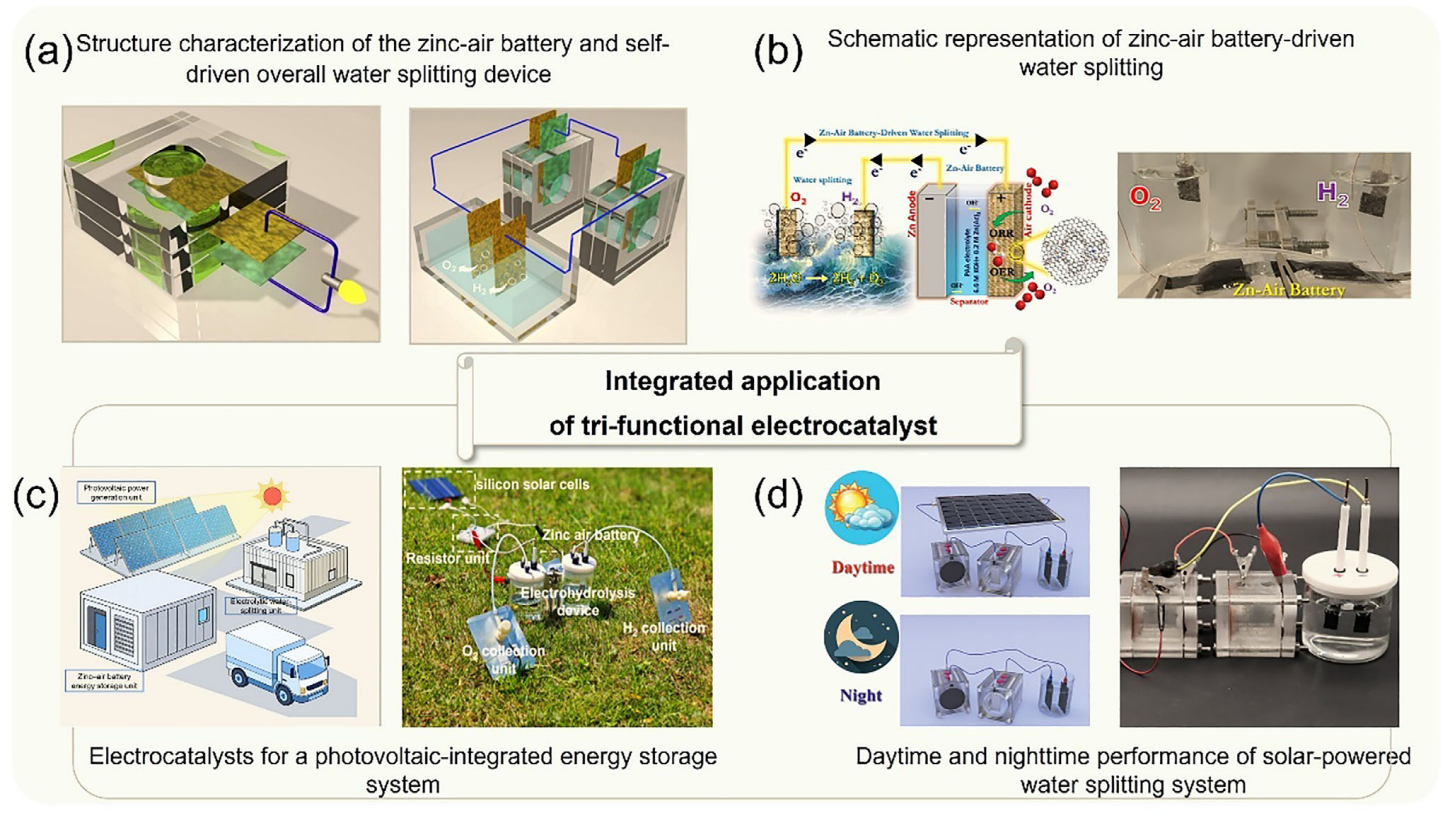
Integrated applications of tri‐functional electrocatalysts in self‐powered energy systems. (a,b) Schematic configurations of ZAB‐driven overall WS systems. Reproduced with permission [143]. Copyright 2019, Elsevier. Reproduced with permission [26]. Copyright 2026, Elsevier. (c,d) Photovoltaic‐assisted energy storage and WS systems for day‐night hydrogen production. Reproduced with permission [29]. Copyright 2025, ACS. Reproduced with permission [144]. Copyright 2021, Elsevier.

At the catalyst level, efforts should be made to further reduce the overpotentials for HER and OER and improve the efficiency of ORR/OER bifunctional air electrodes. This will simultaneously lower the operating voltage of the electrolyzer and raise the output voltage plateau of the ZAB, thereby narrowing the voltage difference between the two [134, 135, 136, 137]. Tri‐functional catalysts capable of stable operation over a wide potential window should be developed to accommodate variations in the discharge voltage of the ZAB and fluctuations in the operating current of the electrolyzer. At the electrode level, self‐supporting integrated electrodes suitable for both ZAB and total WS should be developed. Simultaneously, by regulating pore structure and hydrophilicity/hydrophobicity, it is possible to balance the oxygen diffusion requirements of ZAB with the electrolyte wetting and bubble desorption needs of WS [138, 139]. At the system level, simple voltage management or power regulation modules can be introduced to improve the alignment between the output of the ZAB and the demands of the electrolyzer. Although this increases system complexity and cost to some extent, it helps stabilize the operating current, improve energy utilization efficiency, and extend system lifespan.

Therefore, when evaluating ZAB‐powered WS systems, it is no longer sufficient to rely solely on half‐cell metrics; instead, a system‐level evaluation framework should be established. It is recommended that the following parameters, at a minimum, be reported: the number of ZABs and their connection configuration; discharge curves for individual cells and battery packs; polarization curves of the electrolyzer; actual operating current and voltage of the self‐powered system; continuous operating time; hydrogen and oxygen production rates; Faradaic efficiency; system energy efficiency; and operational stability over multiple cycles. Only through these quantitative metrics can the true contribution and application potential of tri‐functional electrocatalysts in integrated systems be accurately assessed.

The critical parameters discussed above, including ZAB operating voltage, electrolyzer polarization, and energy efficiency systems, are rarely reported together in the literature. To illustrate this data gap, we summarize the available system‐level energy data for representative tri‐functional electrocatalysts in Table 9.

**TABLE 9:** Summary of system‐level energy data for representative tri‐functional electrocatalysts in ZAB‐driven WS systems (2024–2026).

Catalyst	ZAB (V/I) (single cell)	System‐level data						
		Serial number	Electrolyzer (V/I)	H_2_ Rate (µL/s)	Faradaic efficiency	Energy input (J)	Energy output (J)	Refs.
Co@NPCL	OCV = 1.40 V 1.22 V @ 10 mA/cm^2^	/	/	/	/	/	/	[99]
Co_2_P‐NCS‐x	OCV = 1.506 V 1.13 V @ 9.8 mA/cm^2^	2	∼1.7 V 50 mA/cm^2^	3.4	98%	664.4	255.2	[100]
Co_4_N@Fe/N‐C	OCV = 1.38 V 1.21 V @ 2 mA/cm^2^	/	/	/	96.4%	/	/	[101]
Co_9_S_8_‐CoMo_2_S_4_/NSC	OCV = 1.41 V 1.1 V @ 10 mA/cm^2^	2	/	/	1005	/	/	[42]
CoFe@NC/CoFe_2_O_4_/IF	OCV = 1.48 V 1.2 V @ 5 mA/cm^2^	2	/	/	/	/	/	[40]
Co/FeCo‐NCNT/NP	/	/	/	/	/	/	/	[102]
CoFe‐NiFe/NC	OCV = 1.47 V 1.22 V @ 10 mA/cm^2^	2	∼2.4 V	23.5	/	705.6	264.5	[95]
CoMoO_4_/Co_3_O_4_@CC	OCV = 1.43 V 1.2 V @ 10 mA/cm^2^	/	/	/	95.4%	/	/	[89]
CoN NFs	1.2 V @ 10 mA/cm^2^	/	/	/	/	/	/	[103]
Co_2_P/Co‐N‐C	OCV = 1.55 V (liquid) 1.29 V @ 10 mA/cm^2^	3	/	/	/	/	/	[98]
CoP@NC‐Ru	OCV = 1.51 V 0.98 V @ 50 mA/cm^2^	/	/	/	98%	/	/	[104]
CO/R^0^/RO	OCV = 1.54 V	2	/	6.66	98%	/	/	[12]
Co‐S‐INF	0.567 V @ 100 mA/cm^2^	/	/	/	100%	/	/	[96]
CuFe_2_O_4_	1.03 V	/	/	/	/	/	/	[105]
Fe_3_C‐Fe/NC‐800	OCV = 1.46 V	2	/	/	98.4%	/	/	[20]
LaCoO_3_/NiFe LDH	OCV = 1.43 V 0.87 V @ 1.5 mA/cm^2^	/	/	/	/	/	/	[65]
NC@CoNi	OCV = 1.35 V 0.72 V @ 10 mA/cm^2^	/	/	/	/	/	/	[13]
NCMP	OCV = 1.42 V 0.5 V @ 5 mA/cm^2^	/	/	/	/	/	/	[18]
NiFe_2_O_4_/IF	0.77 V @ 10 mA/cm^2^	2	/	/	/	/	/	[106]
NiMOF‐Ni_2_P/IF	OCV = 1.49 V 0.7 V @ 10 mA/cm^2^	/	/	/	/	/	/	[60]
NiO/IrO_2_‐NF	OCV = 1.55 V 0.75 V @ 2 mA/cm^2^	2	/	/	/	/	/	[107]
RuSe_2_CoSe_2_/NC	OCV = 1.52 V	/	1.57 V 10 mA/cm^2^	/	/	/	/	[108]
V_O_‐CoWO_4_	OCV = 1.49 V	/	1.5 V 10 mA/cm^2^	/	/	/	/	[90]
FeNi/FeNiO‐NCS	OCV = 1.56 V	2	1.59 V 10 mA/cm^2^	/	97%	/	/	[93]
FeP/IF	OCV = 1.33 V	2	1.67 V 10 mA/cm^2^	/	/	/	/	[84]
Co_5.47_N/VN@NCFs	OCV = 1.45 V	2	1.53 V 10 mA/cm^2^	/	95.7%	/	/	[37]
Co_9_S_8_/CuCo_2_S_4_	OCV = 1.41 V	2	1.66 V 10 mA/cm^2^	/	/	/	/	[94]
NiOOH/P‐MoS_2_/CC	OCV = 1.46 V	/	1.42 V 10 mA/cm^2^	/	/	/	/	[109]
NCO/CO‐500	OCV = 1.33 V	/	1.72 V 10 mA/cm^2^	/	/	/	/	[14]
CoW‐N/C	OCV = 1.39 V	/	1.79 V 10 mA/cm^2^	/	/	/	/	[64]
RuCu/CoCu/C	/	/	1.51 V 10 mA/cm^2^	/	/	/	/	[110]
FNCO‐600	OCV = 1.55 V	/	1.72 V 10 mA/cm^2^	/	94%	/	/	[33]
TWCH‐Pt/MWCNTs	OCV = 1.56 V	1	PWSV = 2.19 V	/	/	/	/	[29]
g‐C_3_N_4_‐PPy@CNR	OCV = 1.39 V	3	/	/	/	/	/	[71]
Co‐Ni/NiF_2_@PCNFs	OCV = 1.46 V	/	/	/	/	/	/	[39]
FeCo LDH/TiN/NC	OCV = 1.50 V	/	1.57 V 10 mA/cm^2^	/	/	/	/	[44]
GF/Co@NCNTs	OCV = 1.46 V	2	1.59 V 10 mA/cm^2^	16.33	/	/	/	[24]
NiFe‐Co/NC@NMO/NF	OCV = 1.42 V	/	1.54 V 10 mA/cm^2^	/	/	/	/	[111]
NiCo_2_S_4_@GA/NF	OCV = 1.20 V	/	1.68 V 10 mA/cm^2^	/	**/**	/	/	[112]
Mo‐NiFe_2_O_4_/IF	/	2	1.69 V 10 mA/cm^2^	/	/	/	/	[91]
FeNi‐LDH/CA	OCV = 1.54 V	/	1.48 V 10 mA/cm^2^	/	/	/	/	[67]
CoNC_HS_/Cu_NWs_	OCV = 1.43 V	/	1.63 V 10 mA/cm^2^	/	/	/	/	[38]
GOx/N‐CNTs/GCE	/	/	1.60 V 10 mA/cm^2^	/	/	/	/	[113]
Pt/Ni_3_(PO_4_)_2_/NF	OCV = 1.45 V	2	1.58 V 10 mA/cm^2^	/	/	/	/	[24]
Zn‐FeCo@CNF‐900	OCV = 1.47 V	2	1.68 V 10 mA/cm^2^	/	100%	/	/	[19]
Cu_0.81_Ni_0.19_@CoN/Mo_2_N	OCV = 1.36 V	2	1.56 V 10 mA/cm^2^	/	100%	/	/	[36]
FeCoPP/G	OCV = 1.57 V	2	1.61 V 10 mA/cm^2^	/	98.6%	/	/	[26]
NFS‐XC/72	OCV = 1.51 V	/	/	/	/	/	/	[28]

*Notes: E*_input _= *V *× *I *× *t*. Here, *V* is the actual operating voltage of the electrolyzer, as used in the energy input calculation. *E*_output _= nH_2 _× HHVH_2_, HHVH_2 _= 285.8 kJ/mol [140].

Abbreviations: OCV, open‐circuit voltage; PWSV, photovoltaic‐driven water splitting voltage.

### 4.3 Potential for Other Integration Configurations

In addition to the mainstream approach of using ZAB to directly drive WS, tri‐functional electrocatalysts can further enable a variety of novel integrated energy systems, such as solar‐assisted self‐powered systems, flexible wearable systems, and reconfigurable cascaded devices. These new configurations can expand the application boundaries of tri‐functional catalysts and provide new design concepts for distributed energy conversion, portable power supply, and multifunctional device integration.

Solar‐assisted self‐powered systems represent a typical integrated architecture [53, 141], as shown in (Figure 12c,d). In this system, photovoltaic (PV) modules are used during the day to charge the ZAB or directly drive WS, while at night, the ZAB discharges to drive water electrolysis for hydrogen production, thereby achieving temporal decoupling between solar input, energy storage, and hydrogen production. This configuration holds promise for mitigating the intermittency of solar power and enabling round‐the‐clock energy utilization. However, the actual efficiency of this system depends not only on the efficiency of the PV modules and the activity of the catalyst but is also constrained by the matching relationship between the PV output, the battery's charge–discharge characteristics, and the operating voltage of the electrolyzer. Therefore, future research should focus on the overall energy flow through the PV‐battery‐electrolyzer system to quantitatively evaluate the conversion efficiency from solar to hydrogen energy, the stability of the day‐night cycle, and the system degradation mechanisms.

Flexible, wearable, self‐powered systems represent another area of interest. By integrating flexible solid‐state ZAB with flexible electrolytic cells onto a single flexible substrate, it is possible to construct lightweight, bendable, and wearable power supply or hydrogen production systems. In such systems, tri‐functional electrocatalysts must not only exhibit high HER/OER/ORR activity but also maintain excellent mechanical stability, interfacial adhesion, and electrolyte compatibility [106, 142]. For flexible solid‐state ZAB, the ionic conductivity, water retention capacity, and resistance to carbonation of the gel electrolyte significantly impact long‐term operational performance; for flexible electrolyzers, issues such as electrode cracking, catalyst detachment, and impeded gas venting under bending conditions also require systematic investigation. Therefore, the evaluation of flexible integrated systems should encompass both electrochemical performance and mechanical reliability, such as performance retention rates under varying bending radii, bending cycle counts, and ambient humidity conditions.

Reconfigurable cascade devices represent a more conceptual integrated solution. In this configuration, a single electrode or device can switch between battery mode and electrolyzer mode: operating as a ZAB during energy storage and as a water electrolyzer during hydrogen production [62]. This concept holds promise for simplifying device structures and maximizing material utilization, but it also places extremely high demands on catalysts and electrodes. Under different operating modes, the potential window, reactant environment, gas evolution behavior, and interfacial state experienced by the electrode differ significantly. Frequent switching may lead to irreversible restructuring of the active phase, increased interfacial impedance, and mechanical degradation. Therefore, such devices are currently still in the conceptual exploration phase, and their switching efficiency, energy loss, long‐term reliability, and safety require further validation.

In summary, research on tri‐functional electrocatalysts is gradually shifting from the optimization of individual material properties toward a new phase of co‐design involving materials, devices, and systems. However, current research in this area remains largely at the proof‐of‐concept and laboratory demonstration stages. In the future, only by seamlessly integrating the precise regulation of active sites, electrode structure engineering, device performance optimization, and system energy management will it be possible to truly achieve efficient, stable, and scalable ZAB for WS and other integrated energy applications.

## 5 Summary and Perspective

Despite remarkable advances in developing tri‐functional electrocatalysts for WS and ZAB, the field faces a persistent gap between half‐cell heroism and device‐level practicality. This section distills the core scientific and engineering challenges that must be overcome to enable truly integrated, self‐powered energy systems. We then propose a set of actionable research priorities, ranging from the development of unified activity descriptors to the engineering of robust triple‐phase interfaces and the establishment of standardized device testing protocols.

### 5.1 Fundamental Scientific Challenges

From a materials perspective, non‐metal, precious‐metal, transition‐metal, and hydrogen catalysts each offer distinct advantages yet suffer from common underlying limitations. Rather than reiterating specific observations (e.g., stability debates for single atoms or surface reconstruction of sulfides/phosphides), we identify two overarching scientific questions: (1) How can a unified description of tri‐functional active sites be achieved when the structural requirements for HER, OER, and ORR are inherently conflicting? (2) Under operating conditions, how do dynamic restructuring and degradation pathways couple with multi‑reaction selectivity? Addressing these questions requires moving beyond empirical doping and morphology control toward a thermodynamics‑ and kinetics‑guided design framework.

From a mechanistic perspective, the core challenge lies in simultaneously satisfying the distinct intermediate binding energies for HER (H* adsorption/water dissociation), OER (OH*, O*, and OOH* transformation), and ORR (O_2_ activation and four‐electron pathway). A single active site is rarely optimal for all three. Hence, a more general scientific problem emerges: how to design multi‑site synergistic systems where spatial separation, interfacial charge redistribution, and dynamic restructuring achieve functional decoupling and synergistic enhancement across the three reactions.

### 5.2 Device and System‐Level Challenges

From a device application perspective, half‐cell metrics (overpotential, half‐wave potential, Tafel slope) are insufficient. For ZAB, the air electrode must sustain a stable triple‐phase interface under alternating ORR/OER; for WS, electrodes must resist continuous gas evolution and strong oxidizing conditions; for ZAB‐driven WS, voltage/power matching, capacity decay, dynamic load response, and energy efficiency become critical. Therefore, evaluation must expand from half‐cells to electrode, device, and system scales, with standardized protocols that mimic real operating conditions.

Despite significant progress in this field, tri‐functional electrocatalysts still face the following key challenges.

1. Missing unified theoretical framework for tri‐functional active sites. Current empirical strategies (doping, compositing, morphology control) lack predictive power. The fundamental question is: What are the intrinsic descriptors that can simultaneously predict HER, OER, and ORR activity across different material families? Future work should integrate DFT, microkinetic modeling, and machine learning to establish such a framework.
2. Dynamic restructuring and failure mechanisms under operando conditions. Instead of merely noting that transition‑metal sulfides/phosphides reconstruct or that single‑atom stability is debated, the core scientific problem is: How do the actual active phases evolve dynamically as a function of applied potential, electrolyte composition, and reaction time? And how can this evolution be intentionally steered to stabilize tri‐functionality? In situ/operando Raman, XAS, XPS, DEMS, and ETEM should be used to track structural evolution, interfacial reactions, and deactivation pathways‐not just to document known reconstructions, but to uncover predictive stability descriptors.
3. Electrode architecture and device environment as performance determinants. The common observation that powder catalysts work well in RDE but fail in real electrodes points to a deeper issue: How do catalyst layer properties (porosity, binder distribution, hydrophilicity/hydrophobicity, bubble dynamics, oxygen transport) couple with intrinsic activity to determine device‑level performance? Research should shift toward self‑supporting electrodes, hierarchical pores, triple‑phase interface engineering, and robust conductive frameworks, quantifying the gap between intrinsic activity and practical activity.
4. Energy matching in ZAB‑powered WS requires quantitative system analysis. Rather than stating the obvious (operating point determined by polarization curve intersection), the scientific challenge is: How can transient discharge behavior, internal resistance growth, and Faradaic efficiency be dynamically modeled to optimize overall energy efficiency? Future reports must systematically include discharge curves, cell polarization, actual operating current, hydrogen production rate, Faradaic efficiency, overall energy efficiency, and continuous operation time.

### 5.3 Future Perspectives

Looking ahead, the development of tri‐functional electrocatalysts should be advanced in a coordinated manner across the following areas (Figure 13). First, develop atomically precise synthesis strategies, not as a slogan but as concrete methods: single‑atom coordination control, high‑entropy alloy design, heterojunction construction, and metastable phase stabilization. These provide the fundamental active site library. Second, integrate in situ/operando characterization with theoretical calculations to identify actual active phases, key intermediates, and evolution patterns. This feedback loop refines the synthesis strategies from the first step. Third, establish a database spanning composition, structure, synthesis parameters, catalytic performance, stability, and device performance. Use machine learning to predict candidate materials and optimal synthesis conditions, directly feeding into the first two steps. Fourth, move from powder catalysts to membrane electrode assemblies, self‐supporting electrodes, and modular devices. Finally, for ZAB‑driven WS, adopt systems engineering approaches: voltage management, power matching, heat/mass transfer optimization, and long‑term degradation control. The ultimate metric is not catalyst overpotential but overall system energy efficiency under dynamic load.

**FIGURE 13:**
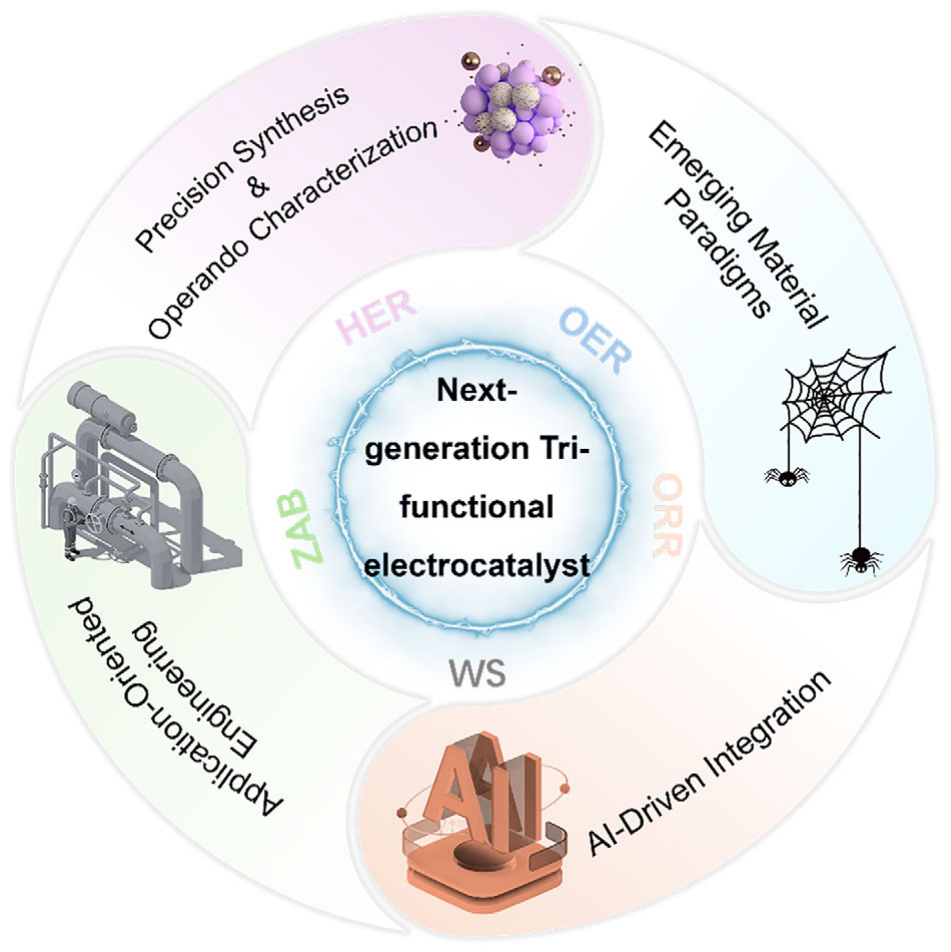
Development trends of next‐generation tri‐functional electrocatalyst.

## Author Contributions

**Xixi Di**: conceptualization, investigation, formal analysis, writing – review and editing, writing – original draft, visualization. **Yichao Wang**: investigation, writing – original draft, writing – review and editing, visualization, validation, formal analysis. **Shuzhen Li**: investigation, writing – original draft, writing – review and editing, visualization, formal analysis. **Chuanyin Xiong**: investigation, writing – original draft, writing – review and editing, formal analysis. **Yonghao Ni**: formal analysis, writing – review and editing, writing – original draft, investigation, conceptualization. **Derek Hao**: conceptualization, investigation, writing – original draft, writing – review and editing, visualization, project administration, supervision, formal analysis. **Yiyang Che**: formal analysis, investigation, writing – original draft, writing – review and editing. **Chao Duan**: conceptualization, investigation, funding acquisition, writing – original draft, writing – review and editing, supervision, formal analysis. **Mengxia Shen**: investigation, writing – original draft, writing – review and editing, formal analysis. **Xiaoshuang Liu**: investigation, writing – original draft, writing – review and editing, formal analysis.

## Funding

The National Natural Science Foundation of China (22178206).

## Conflicts of Interest

The authors declare no conflicts of interest.

## Data Availability

Data sharing not applicable to this article as no datasets were generated or analysed during the current study.
